# Growth Strategies of Tropical Tree Species: Disentangling Light and Size Effects

**DOI:** 10.1371/journal.pone.0025330

**Published:** 2011-09-22

**Authors:** Nadja Rüger, Uta Berger, Stephen P. Hubbell, Ghislain Vieilledent, Richard Condit

**Affiliations:** 1 Spezielle Botanik und Funktionelle Biodiversität, Universität Leipzig, Leipzig, Germany; 2 Institut für Waldwachstum und Forstliche Informatik, Technische Universität Dresden, Tharandt, Germany; 3 Department of Ecology and Evolutionary Biology, University of California Los Angeles, Los Angeles, United States of America; 4 Center for Tropical Forest Science, Smithsonian Tropical Research Institute, Washington, District of Columbia, United States of America; 5 UR105 Forest Ecosystem Goods and Services, Cirad, Montpellier, France; 6 DRP Forêt et Biodiversité, Cirad-Madagascar, Antananarivo, Madagascar; Universita' del Piemonte Orientale, Italy

## Abstract

An understanding of the drivers of tree growth at the species level is required to predict likely changes of carbon stocks and biodiversity when environmental conditions change. Especially in species-rich tropical forests, it is largely unknown how species differ in their response of growth to resource availability and individual size. We use a hierarchical Bayesian approach to quantify the impact of light availability and tree diameter on growth of 274 woody species in a 50-ha long-term forest census plot in Barro Colorado Island, Panama. Light reaching each individual tree was estimated from yearly vertical censuses of canopy density. The hierarchical Bayesian approach allowed accounting for different sources of error, such as negative growth observations, and including rare species correctly weighted by their abundance. All species grew faster at higher light. Exponents of a power function relating growth to light were mostly between 0 and 1. This indicates that nearly all species exhibit a decelerating increase of growth with light. In contrast, estimated growth rates at standardized conditions (5 cm dbh, 5% light) varied over a 9-fold range and reflect strong growth-strategy differentiation between the species. As a consequence, growth rankings of the species at low (2%) and high light (20%) were highly correlated. Rare species tended to grow faster and showed a greater sensitivity to light than abundant species. Overall, tree size was less important for growth than light and about half the species were predicted to grow faster in diameter when bigger or smaller, respectively. Together light availability and tree diameter only explained on average 12% of the variation in growth rates. Thus, other factors such as soil characteristics, herbivory, or pathogens may contribute considerably to shaping tree growth in the tropics.

## Introduction

Growth rates of tropical tree species vary widely between species and in response to resource availability (e.g. light, soil moisture, nutrients) and individual condition (e.g. size, vigor) [1], [2]. Tree growth is an important component of demographic variation among tropical tree species and its response to biotic and abiotic factors reflects different life-history strategies of tropical tree species. An understanding of the drivers of tree growth at the species level is required to predict likely changes of species' abundances and hence community composition and biodiversity [3], [4], as well as to apply detailed process-based simulation models to predict forest dynamics and carbon storage under changing disturbance regimes such as logging or hurricanes [5], [6].

Light availability is widely believed to be one of the most important environmental factors driving growth of tropical rainforest trees [7]-[9]. Tree growth has generally been found to increase with light or gap size [2], [10]-[14]. Species-specific differences in the response of growth to light availability may lead to rank reversals of growth rates along the light gradient and this light-gradient partitioning is believed to contribute to tree species richness [7], [12], [15], [16]. However, studies on limited numbers of tropical tree seedlings have not found evidence of rank reversals between growth rates of different species [1], [17], [18].

Quantifying growth response to light across highly diverse tropical communities is challenging. Many species occur at low densities [19], [20] and measuring the continuous variation in light availability across large spatial scales is labor-intensive [21], [22]. Thus, previous studies have usually focused on a small number of species [9], [23], [24] and the seedling or sapling life stage [10], [16], [25]. Additionally, light availability has been represented indirectly by competition indices based on the density, basal area and/or distance of neighboring trees [26], exposed crown area [9], [24] or disturbance indices [2], but rarely growth has been described as a function of irradiance for tropical tree species (for temperate tree species see [27]–[29]).

As trees grow taller, they experience successively brighter environments [30], [31]. This correlation between light availability and tree size has to be taken into account and the effects of light and size have to be separated [32]. There are few studies that have included measures of tree size and light availability simultaneously as predictors of tree growth, and they consistently found significant effects of both variables for the majority of species [2], [24], [26].

In this study, we aim to disentangle the effects of light availability and tree size on growth across >90% of the 300 tree species occurring at Barro Colorado Island (BCI), Panama. Forest census data from the 50-ha Forest Dynamics Plot at BCI provided information on the spatial location, size and diameter growth of nearly 150 000 individual trees of 274 species in two census intervals (1985–1990, 1990–1995). Yearly canopy census data that recorded vegetation density in six height layers were used as a proxy of light availability for each individual tree.

We used a hierarchical Bayesian approach to quantify the response of growth to light availability and tree size across the entire community, including rare species. Hierarchical models address the differences in sample size between the species by combining probability models for the growth variability within species and the variation between species [20], [33]. In our two-level hierarchical model, individual growth is a function of light and size, while species-level parameters are related to a species' abundance [34]. This allows us to estimate the distribution of species-specific light and size effects on growth and their relationship with abundance across the community, properly discounting information on rare species relative to common. The Bayesian approach also allows correctly accounting for process and measurement error, e.g. including negative growth observations [35].

Here we report (1) the distribution of growth rates at standardized conditions (5 cm dbh, 5% light), light and size effects on growth across the community to assess the degree of growth strategy variation between species. For abundant species (≥100 stems) parameter estimates are largely determined by the data on the given species rather than by the community-level information. Therefore, (2) we use parameter estimates of abundant species to evaluate the respective contributions of light availability and tree size to growth rates, and the scope for light gradient partitioning.

## Methods

### Study area

We analyzed data from a 50-ha forest census plot on BCI, Panama (9°9′N, 79°51′W). BCI is a 1567-ha island in the Panama Canal covered with tropical lowland moist forest. The plot consists of 48 ha of undisturbed old-growth forest and 2 ha of secondary forest about 100 years old [36]. The climate on BCI is warm throughout the year, but rainfall is seasonal with most of the 2500 mm falling during the wet season from April to November [37], [38]. Elevation of the plot is 120–155 m asl [39]. Detailed descriptions of flora, fauna, geology and climate can be found in [40]–[42]. Barro Colorado Island is managed exclusively for field research by the Smithsonian Tropical Research Institute (STRI), which has been granted long-term custodianship over the island by Panama's Environmental Authority. STRI gave permission to establish the 50-ha plot as a permanent census in 1980.

### Growth data

All free-standing woody stems ≥1 cm diameter at breast height (dbh) were mapped, identified to species and measured in 1981–1983, 1985, and every 5 years thereafter (www.ctfs.si.edu; [39], [43]). Here we use the census intervals from 1985–1990 and 1990–1995 and determined annual dbh growth rate (mm/yr). We discarded cases where a tree survived but its stem was measured at a different height, or where one stem broke so a resprouted stem of the same tree was measured. We also excluded outliers: stems which grew >75 mm/yr or shrunk >25% of their initial dbh. Smaller negative growth observations due to dbh measurement error were included (see Estimation of measurement error). Due to their lack of secondary growth, we excluded palm species. Because dbh values were rounded down to the nearest mm for all stems <55 mm in 1985 but not in 1990, it was necessary to round 1990 dbh values below 55 mm down as well before calculating growth rates. Rounding down may bias growth estimates of small stems. However, we found that the bias introduced by rounding down is minimal. On average, growth is underestimated by 0.03 mm/yr (cf. [44]). Growth rates for the second census interval are based on dbh measurements with 1 mm accuracy. To avoid edge effects of the light availability calculation, we excluded all individuals within 20 m of any edge of the plot. In total, 144 967 individuals of 265 species and 148 989 individuals of 270 species were included in the analysis in the first and second census interval, respectively.

### Estimation of measurement error

To estimate the error of dbh measurements, 1562 randomly chosen trees were measured twice within 30 days. Assuming that trees did not grow between these measurements, we applied a Bayesian model to estimate true dbh for each tree and fit the differences between measured dbh and true dbh with a sum of two normal distributions [45]. The first describes small errors that are proportional to the dbh of the tree and has a s.d. of *SD_1_ =  sda+sdb×dbh*. The second is independent of tree dbh and describes larger errors, e.g. due to errors in decimal places or recording dbh for the wrong tree and has s.d. *SD_2_*. Errors were best fit with *sda*  =  0.927 mm (s.d. of the posterior distribution was 0.024 mm), *sdb*  =  0.0038 (s.d. = 0.00036), *SD_2_* = 25.6 mm (s.d. = 2.49 mm), with a fraction (*f*) of 2.76% (s.d. = 0.39%) of the trees being subject to the larger error. Growth calculations involve two dbh measurements, thus the variance of growth error is twice the variance of measurement error. The posterior distributions of error parameters *sda*, *sdb*, *SD_2_* and *f* enter as fixed priors in the hierarchical Bayesian model.

### Estimation of light availability

We used annual canopy census data to produce an index of the amount of light reaching any point in the forest. The censuses were conducted from 1983 to 1996, except for 1994. Thereafter, the canopy census was omitted for several years and then continued applying a different method. Thus, consistent canopy census data are only available for the two census intervals 1985–1990 and 1990–1995, and we restricted our analysis to these two intervals. The canopy census recorded the presence of vegetation in six height intervals, 0–2, 2–5, 5–10, 10–20, 20–30 and ≥30 m every 5-m across the 50 ha. For each tree, we calculated a shade index as a weighted sum of vegetation located above the tree and <20 m away.

Light measurements were not available for any tree in the study area, i.e. we could not calibrate the shade index directly. Instead, we used 396 direct measurements of relative irradiance at a nearby site on BCI in 1993 [46] and converted the shade index to an estimate of relative irradiance by fitting a nonlinear regression through the 5^th^, 25^th^, 50^th^, 75^th^ and 95^th^ percentiles of the two distributions. This approach made it impossible to estimate the measurement error for the light index. However, the light index performed well in predicting recruit numbers across 263 species at BCI [47]. Details about its calculation are given in [47], [48]. Even though dbh and the light estimate are strongly correlated (cor = 0.8), there are many observations of small trees receiving high light and of larger trees receiving low light (Fig. S1). This allows us to separate the effects of the two variables.

### Variable selection

Because the hierarchical Bayesian model requires long computation times, we first performed independent regressions of log(growth) as a function of dbh and light to select the best functional relationship. Stems with negative growth were excluded from this analysis. We applied a step-wise procedure to determine the best predictors of log(growth) for 98 species with >100 individuals and used AIC [49] to compare models of different complexity. Log(light) was the best predictor of growth, significantly improving the model (|ΔAIC|>2) compared to a constant model for 76 species. Including log(dbh) significantly improved the model further for 64 species. Including dbh or an interaction between log(light) and log(dbh) improved the model significantly for 50 species and 43 species, respectively. However, these more flexible models predicted artificially low or high growth at large dbh in some species, and we decided to adhere to the simpler model only including log(light) and log(dbh) as predictors. Including dbh or an interaction term did not alter the conclusions of this study.

### Hierarchical Bayesian model

To assess the light and size dependence of growth across tree species at BCI, we used a hierarchical Bayesian framework [33], [50] which allows including different sources of error [35]. At the core of the model is the functional relationship predicting growth of individual *i* (*pred_i_*) of species *j* as a power function (linear log-log relationship) given light availability and dbh,



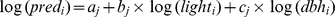
where parameters *a_j_*, *b_j_* and *c_j_* describe the mean growth rate, the light and size dependence of growth of species *j*, respectively.

Variation of growth at a given light availability and dbh is modeled using a lognormal distribution (process error)



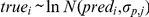
where *true_i_* is the estimated true growth rate of tree *i*. The process error (*σ_p_*) is estimated for each species. Using a lognormal distribution, the process error automatically scales with predicted growth [51].

Data enter our model as the observed annual dbh growth of individual *i* (*obs_i_*, mm/yr) and is assumed to be subject to measurement error as described above



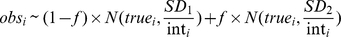
 with *SD_1_* describing the size-dependent error component and *SD_2_* the size-independent error component affecting *f* = 2.7% of the observations. Standard deviations have to be adjusted to the time period elapsed between the two dbh measurements of the tree (*int_i_*) from which the annual growth rate has been calculated.

Preliminary analyses indicated that *a_j_*, *b_j_*, and *c_j_* varied slightly but systematically with abundance across the community. Therefore, we included abundance (*abun_j_*) as species-level predictor of parameters of the growth model. Abundance is measured as the number of individuals ≥1 cm dbh in the 50-ha plot that survived the given census interval. Non-hierarchical model runs also revealed that all model parameters were approximately normally distributed at a given abundance. Thus,



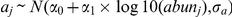





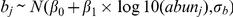





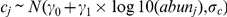



The standard deviations *σ_a_*, *σ_b_*, and *σ_c_* measure the between-species variation. As we did not have prior knowledge, we used non-informative uniform priors for these hyperparameters:



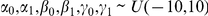





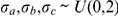



The process error (*σ_p_*) was assumed to vary lognormally across the community with hyperparameters *µ_h_* and *σ_h_*. Priors for both parameters were



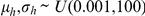



The model assumes no correlation between parameters.

### Model implementation and diagnostics

Posterior distributions of the species-specific parameters of the growth model, true growth of each individual tree, error components and hyperparameters were obtained using a Markov chain Monte Carlo (MCMC) method that is a hybrid of the Metropolis–Hastings algorithm and the Gibbs sampler [20], [52]. Parameter values are sequentially updated as in the Gibbs sampler, but acceptance depends on the likelihood ratios as in the Metropolis–Hastings algorithm [53]. The proposal distribution is a normal distribution centered on the current value of the given parameter. The step width for each parameter, i.e. the standard deviation of the proposal distribution, is constantly adjusted during the burn-in period in such a way that acceptance rate is kept around 0.25 [53].

To speed up the convergence of the Gibbs sampler, we weakened the correlation of *a* with *b* and *c* by centering the light and dbh data on approximately median or mean values across all individuals



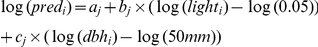



Median light is 0.045 and mean dbh is 45 mm. Thus, *a_j_* represents the log of predicted annual growth of a tree with 5 cm dbh that receives 5% light.

We monitored convergence by running two chains with different initial values and used the Gelman and Rubin's convergence diagnostics and a value of 1.1 to detect convergence [52], [54]. *Post-hoc* analyses showed that the correlation between model parameters was <|0.57| for all parameter combinations. These correlations were not strong enough to prevent the chains from mixing well and convergence required 100 to 2900 iterations. We used a burn-in period of 5 000 iterations and additional 5 000 iterations were used for analysis. We computed posterior parameter distributions given observed growth, light availability and dbh of each individual. All analyses were carried out using the software package R version 2.11.1 [55] and simulations were run on parallel computers.

### Analysis

From the posterior distributions, we computed the mean, s.d. and 95% credible intervals (CI) of all model parameters. Significance of coefficients was assessed by a 95% CI that did not include zero. For abundant species with >100 individuals, model parameters and CIs are largely determined by the data available for the given species. For less abundant species, parameter estimates are increasingly determined by the species-level prediction based on abundance and CIs are constrained by the between-species variation (*σ_a_*, *σ_b_*, *σ_c_*).

To report predicted annual mean growth rate at 5 cm dbh and 5% light, we back-transformed *a_j_* to arithmetic units: 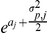. Likewise, to compare model predictions with data, we back-transformed predicted growth rates on the logarithmic scale (*pred_i,l_*) to the arithmetic scale (*pred_i,a_*),



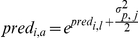
and plotted observed and predicted growth rates for each individual tree as well as mean observed and predicted growth rates in different size and light classes, respectively. For species with <25 individuals, all individuals were pooled.

To evaluate between-species variation of growth rates of rare and abundant species at different light levels, we calculated the probability density of median annual growth rate 
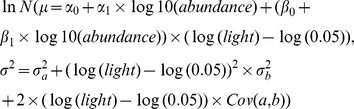
from the hyperparameters of *a* and *b* at low (N = 10) and high (N = 1000) abundance, and low (5%) and high (20%) light, respectively. For these calculations we used hyperparameters estimated for the census interval 1985–1990. The covariance between *a* and *b* was 0.033 and 0.025 for rare and abundant species, respectively.

To visualize the effect of light on growth, we calculated mean observed and predicted growth rates in ten light classes corresponding to deciles of light observations. Predicted mean growth rates were calculated at mean observed light level and mean dbh of the individuals in the respective light class. To visualize the effect of dbh on growth, we calculated mean observed and predicted growth rates in different size classes. The number of size classes depended on the abundance of the species. Predicted growth rates were calculated at mean observed light level and mean dbh of the individuals in the respective size class.

The percentage of explained variance (R^2^) was evaluated as
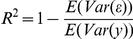

[50]. This measure can be negative when the model predicts the data so poorly that residual variance is larger than the variance of the data.

To (visually) assess the conservation of growth rankings among species at different light conditions, we computed average growth for species with ≥100 individuals over the range of most commonly observed light conditions (2–20%) from the parameters estimated for the respective census interval. Average tree dbh at the different light levels was determined from a nonlinear regression predicting log(dbh) using log(light) and light as predictors across all individuals (Fig. S1). We also computed Spearman's rank correlation (rho) for growth rates at 2% and 20% light.

For species with ≥100 individuals, we calculated contributions of light and dbh to growth by comparing growth rates at standardized conditions. For saplings, we calculated the difference between predicted growth at baseline conditions defined as 1 cm dbh and average light availability at 1 cm dbh (2%) and growth when light and dbh are doubled, i.e. 2 cm dbh and 4% light. In this analysis, only species with maximum dbh ≥2 cm were included. For larger trees (only for species with maximum dbh ≥10 cm), we performed similar comparisons using growth at 10 cm dbh and average light availability (18%) as a baseline and comparing it to growth at doubled dbh (20 cm) and doubled light (32%).

## Results

### Community-level patterns of growth

**Figure 1:**
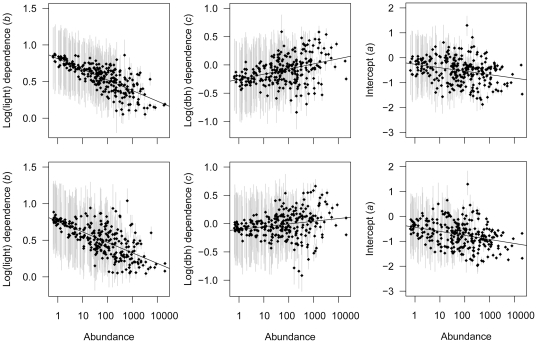
Parameter estimates of the growth model. Means (points) and 95% credible intervals (lines) of the species-specific parameters of the growth model. Left panels: log(light) dependence (*b*), middle panels: log(dbh) dependence (*c*), and right panels: intercept (*a*) for 265 tree species in the census interval 1985–1990 (upper panels) and 270 species in the census interval 1990–1995 (bottom panels) at BCI, Panama. Sensitivity of growth to light (*b*) and growth at standardized conditions (*a*) decrease significantly with abundance. Abundance is slightly jittered to reduce overlap of rare species.

All species grew faster at higher light (*b*>0; Fig. 1). This effect was significant for the majority of species; 98% and 93% in the two census intervals, respectively. However, between-species variation in light response was limited. All but two species' parameter estimates and most of the CIs were between 0 and 1, indicating a decelerating increase of growth with light (Fig. 1; Table S1). A significant portion of between-species variation in light response was explained by a species' abundance, with abundant species responding less strongly to changes in light availability (Table 1; Fig. 1).

**Table 1:** Parameters of the species-level model of tree growth. Means (s.d.) of parameter estimates from the species-level model. Coefficient estimates that are significantly different from zero (based on 95% credible intervals) are highlighted in bold.

Model coefficient	Intercept*α_0_, β_0_,* γ*_0_*		Abundance*α_1_*, *β_1_*, γ*_1_*		Standard deviation*σ_a_*, *σ_b_*, *σ_c_*	
	1985–1990	1990–1995	1985–1990	1990–1995	1985–1990	1990–1995
Intercept (*a*)	**−0.261** (0.128)	**−0.437** (0.095)	**−0.139** (0.053)	**−0.162** (0.041)	0.558 (0.034)	0.565 (0.033)
Log(light) (*b*)	**0.812** (0.100)	**0.756** (0.058)	**−0.145** (0.037)	**−0.143** (0.023)	0.184 (0.015)	0.226 (0.018)
Log(dbh) (*c*)	**−0.255** (0.095)	**−**0.102 (0.083)	**0.091** (0.036)	0.048 (0.033)	0.234 (0.019)	0.301 (0.020)

Growth rate also varied with tree size (*c*), but increases were as common as decreases (Fig. 1; Table S1). Only few of the species' parameter estimates were significantly positive (12% in both census intervals) or negative (12% in the first and 8% in the second census interval; Fig. 1; Table S1). Abundant species showed growth increases with size more often than rare species, but this relationship was only significant in the first census interval (Table 1).

**Figure 2:**
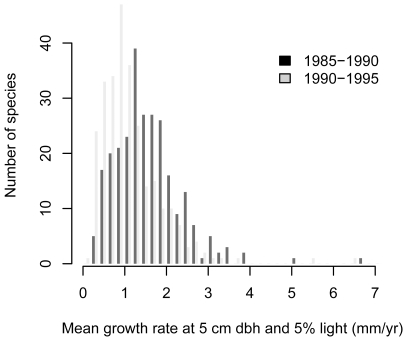
Growth rates at standardized conditions. Predicted annual growth rate (mm/yr) at 5 cm dbh and 5% light for 265 tree species in the census interval 1985–1990 and 270 species in the census interval 1990–1995 at BCI, Panama.

The intercept of the growth model (*a*) was weakly but significantly related to abundance, with rare species tending to grow faster than abundant species (Table 1; Fig. 1). Back-transformed to the arithmetic scale, average growth rates at standardized conditions (5 cm dbh and 5% light) varied widely between species and ranged from 0.2 to 6.7 mm/yr (Fig. 2). Average growth (at standardized conditions) was 1.6 mm/yr in the first census interval and 1.2 mm/yr in the second interval.

**Figure 3:**
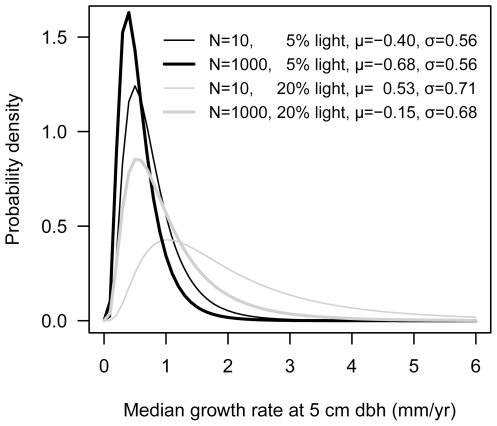
Distribution of median growth rates for rare and abundant species under different light conditions. The probability distribution of median growth rate (mm/yr) at 5 cm dbh for rare (N = 10) and abundant (N = 1000) species at low (5%) and high (20%) light is calculated from the hyperparameters of *a* and *b* for the census interval 1985–1990.

Median annual growth rates of 95% of the species are expected to be 0.22–2.0 mm/yr for rare species (N = 10) at low light (5%) (Fig. 3). This is a 9-fold variation. For abundant species (N = 1000) at low light, this range is 0.17–1.5 mm/yr (9-fold variation). Under high light (20%), the predicted ranges are 0.43–6.8 mm/yr (16-fold variation) and 0.22–3.3 mm/yr (15-fold variation) for rare and abundant species, respectively. Thus, the range of median growth rates is almost twice as large under high light (20%) compared to low light (5%). This difference is entirely due to the slightly larger s.d. at higher light.

### Observed versus predicted growth

**Figure 4:**
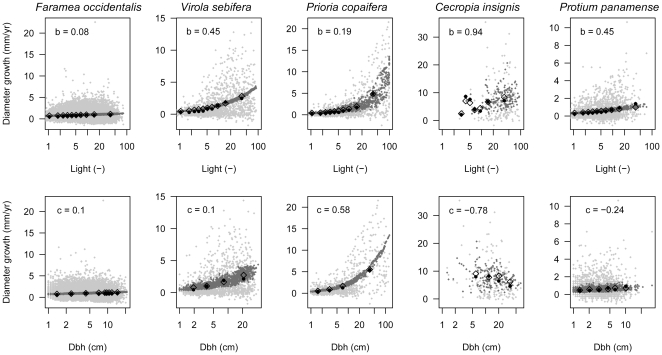
Observed and predicted growth rates against light availability and stem size. Light (*b*) and dbh dependence (*c*) of growth rates for five species in the census interval 1990–1995. Observed (dark grey) and predicted (light grey) growth rates of individuals, mean observed (filled circles) and predicted (diamonds) growth in different light and dbh classes. Species' parameters are given in the panels. Species are *Faramea occidentalis* (N = 20 110), *Virola sebifera* (N = 1469), *Prioria copaifera* (N = 979), *Cecropia insignis* (N = 164) and *Protium panamense* (N = 2227).

We illustrate different combinations of growth response to light and dbh (Fig. 4). Note that x-axes are on log scale and thus, growth increases that are slower than linear (0<*b*<1 or 0<*c*<1) appear exaggerated. The shade-tolerant understory tree *Faramea occidentalis* grew slowly and neither light nor dbh explained much of the variation in growth rates. Growth of the shade-tolerant midstory tree *Virola sebifera* responded strongly to light, whereas the effect of dbh was insignificant. For *Prioria copaifera*, a shade-tolerant canopy tree, dbh had a large positive impact on growth, while it responded only moderately to light. Growth of the light-demanding pioneer tree *Cecropia insignis* increased strongly at higher light, and at the same time strongly decreased with dbh. Growth of *Protium panamense*, a shade-tolerant midstory tree, decreased with dbh and the apparent slight increase of growth at larger dbh was due to the strong positive effect of light. Model fits for the other species are provided as supporting information (Table S2; Figs. S2, S3).

### Conservations of growth rankings

**Figure 5:**
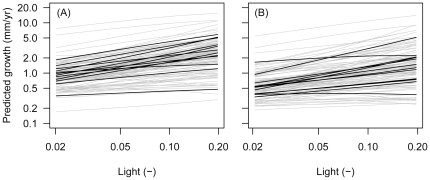
Visual assessment of the scope for light gradient partitioning. Predicted average growth rates (mm/yr) in (A) 1985–1990 and (B) 1990–1995 across most common light conditions (2–20%) for species with ≥100 individuals. Twelve randomly selected species are highlighted in black. Intersections of lines represent reversals of growth rankings between species.

Combining the estimated parameters, we predicted average tree growth for 115 and 114 species with ≥100 individuals in the two census intervals, respectively, across the most common light conditions (2–20%; Fig. 5). Average growth curves ran largely in parallel and growth rankings were preserved to a large degree among the species. Rank correlations (Spearman's rho) between predicted growth rates at 2% and 20% light were 0.69 and 0.66 in the two census intervals, respectively.

### Contributions of light and size to growth

**Figure 6:**
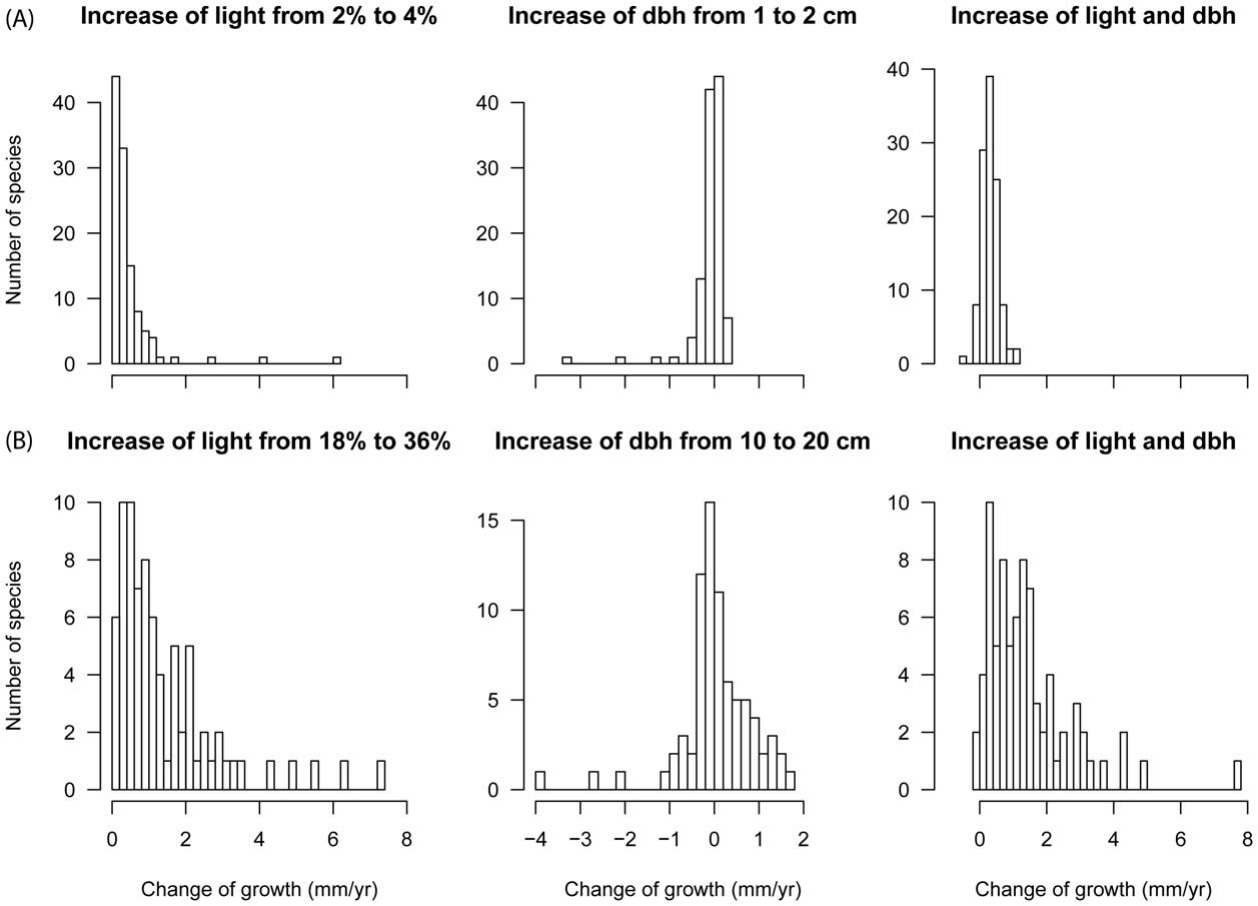
Impact of light availability and stem size on growth rates. Contributions of light availability and size to growth for species with ≥100 individuals. For small trees (A), contributions are expressed by the absolute difference between baseline growth rate of a tree at 1 cm dbh and average light (2%) and growth when light is doubled (4%), dbh is doubled (2 cm) or both. For larger trees (B), contributions are expressed by the difference between baseline growth of a tree at 10 cm dbh and average light (18%) and growth when light is doubled (36%), dbh is doubled (20 cm) or both.

Doubling the light level for small trees of 1 cm dbh led to an increase of growth that was <1 mm/yr for the majority of species with ≥100 individuals (Fig. 6). Doubling the dbh, either led to faster or slower growth, but the effect was small (<0.5 mm/yr). Doubling light and dbh, most trees grew up to 1 mm/yr faster. For larger trees (10 cm dbh), effects were larger because baseline growth rates were larger. Doubling the light level led to an increase of growth that was <3 mm/yr for the majority of abundant species. Doubling the dbh, growth either increased or decreased by up to 2 mm/yr. Doubling light and dbh, the majority of species grew up to 4 mm/yr faster.

### Uncertainty and explained variance

The process error (*σ_p, j_*) varied little across species and between census intervals; *µ_h_* was 1.28 in the first census interval and 1.15 in the second, *σ_h_* was 0.09 and 0.16 in the two census intervals, respectively. Thus, at predicted growth of 1 mm/yr, the probability of true growth being between 0 and 2 mm/yr was 71%. When predicted growth was 5 mm/yr, the probability that true growth was between 3 and 7 mm/yr was 26%.

Dbh and light explained a low proportion of the variation in growth rates. R^2^ was <0.5 for the majority of species and reached 12% and 13% on average in the two census intervals, respectively. For 94 and 92 species in the two census intervals, R^2^ was negative indicating that the model predicted the data so poorly that residual variance was larger than the variance of the data. The majority of these species had <30 individuals.

## Discussion

### Light effect on growth and light gradient partitioning

For the first time it was possible to disentangle light and size effects on growth across a diverse community of tropical tree species. All species grew faster at higher light availability. This effect was significant for >90% of the species. This result confirms that light is effectively an important limiting resource for woody species in natural forests, not only at the seedling and sapling life stage (cf. [56] for temperate trees). However, the low proportion of variation of growth rates that is explained suggests that other factors considerably contribute to shaping tree growth in the tropics. We attribute the larger proportion of significant results – as compared to previous studies [2], [24], [57] – to the larger sample sizes and the higher precision of the light index derived from the small-scale canopy census in capturing actual light availability.

However, we found little evidence for light gradient partitioning in terms of growth rates among species. The majority of species showed a decelerating increase of growth with light (0<*b*<1). Moreover, correlations of growth ranks at 2% and 20% light were high (Spearman's rho ∼ 0.7). The consistency in the response of growth to light across species suggests that reversals in rank order do not have a strong impact on the outcome of competition among species. Largely consistent growth rankings are also reported for seedlings of small numbers of tropical tree species across different light [1], [17], [58] or soil/water treatments [18] or both [59]. Studies that suggest the possibility of light gradient partitioning are often based on very few species [11], [60].

On the other hand, growth rates vary twice as much among the species at high light (16-fold) as compared to low light (9-fold). These large differences in growth rates certainly offer opportunities for differentiation of life-history strategies. Fully understanding the importance of differences in growth rates and light response for coexistence, however, requires the application of simulation models that combine information on the response of all demographic rates (recruitment, growth and mortality) to changes in light availability. The parameters we have estimated here provide the basis for such modeling studies.

The fact that an interaction between light and size improved the model only in 43 species (16%) suggests that species respond in a similar way to changes in light availability across all sizes. This conclusion is corroborated by separate analyses for trees smaller and larger than 5 cm dbh where the estimated distribution of light response was very similar (results not shown). This is in contrast to a study on *Nothofagus solandri* var. *cliffortioides*, where only small trees <10 cm dbh were sensitive to changes in above-ground competition [61].

### Size effect

While species responded consistently to light, changes of diameter growth with tree size were very variable among species with about half the species growing faster when taller and the other half of the species growing faster when small. Likewise, significant responses were as frequently positive (12%) as negative (8%–12%). However, we caution against a physiological interpretation of size dependence of diameter growth rates. If diameter growth scales with diameter as dg ∼ d^c^ and biomass scales with diameter as b ∼ d^8/3^ [62] then biomass growth scales with diameter as bg ∼ d^5/3+c^. Constant biomass growth is achieved when *c* = −5/3. When *c*>−5/3, biomass growth increases with diameter, despite possibly decreasing diameter growth. Parameter estimates and credible intervals of *c* indicate that no species is expected to have *c*<−5/3. This suggests that tropical trees continue accumulating carbon at increasingly faster rates as they grow [4], [63].

For the vast majority of species, growth rates were well approximated by the power function which only allows for a monotonic increase or decrease of growth with diameter. Many other studies have allowed for a hump-shaped response of growth to diameter [2], [24], [26], [64]–[66]. However, we found little evidence for a peak of growth rates at intermediate diameters. It is only a small proportion of species which show this phenomenon including *Cordia alliodora*, *C. bicolor*, *Hirtella triandra*, *Jacaranda copaia*, *Protium tenuifolium*, and *Zanthoxylum ekmanii*.

In other species, the model did not capture the saturation of growth rates at large diameters (e.g. Alseis blackiana, Dipteryx oleifera, Inga marginata, Ocotea oblonga, O. whitei, Pouteria reticulata, Prioria copaifera, Quararibea asterolepis, Simarouba amara). This is a result of the unbalanced data sets dominated by the many small individuals. Nevertheless, the model fits show that for the vast majority of species, growth rates were well approximated and deviations only affected the largest size classes. To account for unbalanced data sets and unknown functional relationships between predictor variables and growth, semi- or nonparametric approaches could be used. However, semi- or nonparametric approaches are not suitable for our study because they prevent a straightforward comparison among many species.

### Uncertainty and unexplained variance

Generally, a large variation of growth rates is observed that is not explained by tree size or light availability [4]. In our analysis, the two factors explained on average across species 12–13% and at most 64% of the variance of growth rates. In faster-growing species, R^2^ was slightly higher than in slow-growing species [67]. These values are in the range of other studies on tropical trees using diameter and crown illumination index [24] or a species-specific neighborhood competition index [26] as predictors of tree growth. In temperate trees, larger values of R^2^ have been reported. Using tree size and exposed crown area or accounting for above and belowground competition, up to 90% of growth variance of North American tree species could be explained [66], [68].

The large amounts of unexplained variance can be due to environmental conditions not considered here, e.g. soil texture [24], soil moisture [67], [69], nutrient availability [18], [70], below-ground competition [61], soil biota [71], year-to-year variation in cloud cover or temperature [23], [35], the identity of neighbors [66], [67], [72], or genetic variability within species [35]. In the study area, the elaboration of fine scale maps of soil nutrients and moisture is underway, and these may lead to further understanding of variation in growth.

Uncertainty in our model fits also comes from other sources: error in the light estimate, measurement error of past growth, and sampling error in rare species [23], [35], [73]. Our light index is only an approximate way of estimating the light environment in the forest. However, measuring light at every tree in the 50-ha plot would involve a prohibitive amount of time and labor. Until LiDAR-mapping data of the entire 50 ha are available, the method we propose offers an objective and straightforward measure of how much vegetation is blocking the sky above any tree of any height in the entire forest. In a previous study based on the same estimation of light availability, a strong impact of light on species-specific recruitment rates was detected [47]. This indicates that our light index captures relevant spatial heterogeneity of light availability.

### Future directions

A problem inherent in highly diverse tropical forests is the low number of individuals per area of the many rare species [19]. Hierarchical Bayesian methods explicitly account for this problem by superimposing a form of variation of the studied phenomenon across the community and by including species-level predictors to constrain parameter estimates of rare species. We used the relationship between a species' abundance as a species-level predictor and found that it explained a small but significant portion of growth parameters. Rare species tended to grow faster and showed a greater sensitivity to light than abundant species.

However, functional characteristics of tree species such as wood density, specific leaf area or maximum height may relate more directly to growth strategies and hold greater promise for a more mechanistic understanding of life-history strategies of tropical tree species [56], [74]. Therefore, as a next step, we aim to include information on species' functional traits to explore their capacity to predict recruitment, growth, and mortality rates as well as the sensitivity of these demographic rates to changes in resource availability. Based on such relationships, studies on recruitment, growth and mortality could be integrated into dynamic simulation models to further investigate the consequences of species differences with respect to demographic characteristics for species coexistence in highly diverse tropical forests.

## Supporting Information

Figure S1 **Diameter-light relationship.** (A) Light estimate vs. tree diameter (dbh) for 148 933 trees at Barro Colorado Island, Panama, in 1990. Trees with dbh >1 m are assumed to receive full sunlight and are not shown. (B) Nonlinear regression predicting average log(dbh) in the light range from 2 to 20% (log(dbh)  =  4.547+0.455×log(light)+2.006×light; dbh is in mm). Average log(dbh) is used to estimate average growth across the light range for Fig. 4.
(PDF)

Figure S2 **Light dependence of growth rates for species with ≥25 individuals in the two census intervals (1985−1990, 1990−1995).** Light classes correspond to deciles of light availability across all individuals. Predicted mean growth rates were calculated at mean observed light level and mean dbh of the individuals in the respective light class. Observed and predicted growth rates of individual trees are displayed as grey and orange dots, respectively. Mean observed and predicted growth rates in different light classes are displayed as black and red dot, respectively.
(PDF)

Figure S3 **Dbh dependence of growth rates for species with ≥25 individuals in the two census intervals (1985−1990, 1990−1995).** Observed and predicted growth rates of individual trees are displayed as grey and orange dots, respectively. Mean observed and predicted growth rates in different size classes are displayed as black and red dot, respectively. For species with <100 individuals, the dbh range was split into three size classes each containing a third of the individuals. For species with <2000 individuals, four size classes contain 25% of the individuals each. For species with <3000 individuals, six size classes contain 25%, 25%, 25%, 15%, 5% and 5% of the individuals, respectively. For species with <4000 individuals, seven size classes contain 25%, 25%, 25%, 15%, 3.3%, 3.3% and 3.3% of the individuals. For species with ≥4000 individuals, nine size classes contain 25%, 25%, 25%, 15%, 2%, 2%, 2%, 2% and 2% of the individuals. We only plot the size dependence for species with a maximum dbh of >3 cm. Predicted mean growth rates were calculated at mean observed light level and mean dbh of the individuals in the respective size class.
(PDF)

Table S1 **Posterior means, lower and upper limits of 95% credible intervals (CI−, CI+) of the average growth rate at 5 cm dbh and 5% light (** ***a*** **), log(light) dependence (** ***b*** **), log(dbh) dependence (** ***c*** **), and process error (** ***σ_p_*** **) for two census intervals (1985–1990, 1990–1995) and tree species in the 50-ha Forest Dynamics Plot at Barro Colorado Island, Panama.** Dashes indicate no living individuals at the beginning of the respective census interval.
(XLSX)

Table S2 **Observed and predicted average growth rate for tree species with <25 individuals at Barro Colorado Island, Panama.** N is the number of individuals.
(XLSX)
