# Supplementary material for: Growth Strategies of Tropical Tree Species: Disentangling Light and Size Effects
Source: PLoS One. 2011 Sep 22;6(9):e25330. doi: 10.1371/journal.pone.0025330 (PMC3178650; doi:10.1371/journal.pone.0025330)
Supplement: Figure S1 — Diameter-light relationship. (A) Light estimate vs. tree diameter (dbh) for 148 933 trees at Barro Colorado Island, Panama, in 1990. Trees with dbh >1 m are assumed to receive full sunlight and are not shown. (B) Nonlinear regression predicting average log(dbh) in the light range from 2 to 20% (log(dbh) = 4.547+0.455×log(light)+2.006×light; dbh is in mm). Average log(dbh) is used to estimate average growth across the light range for Fig. 4. (PDF) [file pone.0025330.s001.pdf]

Figure S1

Diameter-light relationship

(A) Light estimate vs. tree diameter

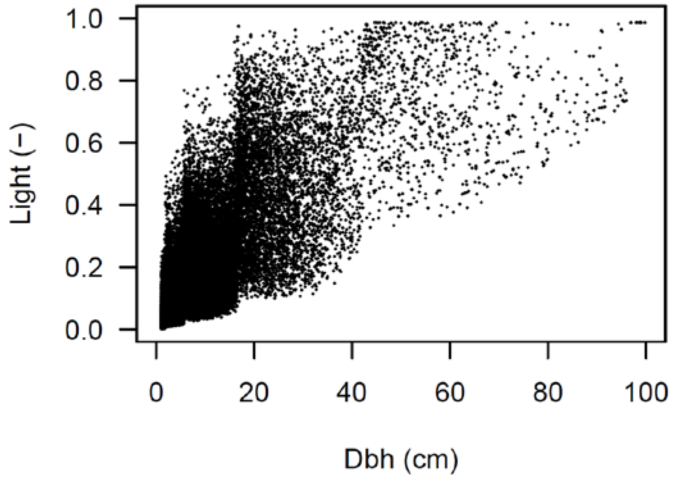

Light estimate vs. tree diameter (dbh) for 148 933 trees at Barro Colorado Island, Panama, in 1990. Trees with dbh > 1 m are assumed to receive full sunlight and are not shown.

(B) Nonlinear regression estimating average tree dbh for light levels between 2 and 20%

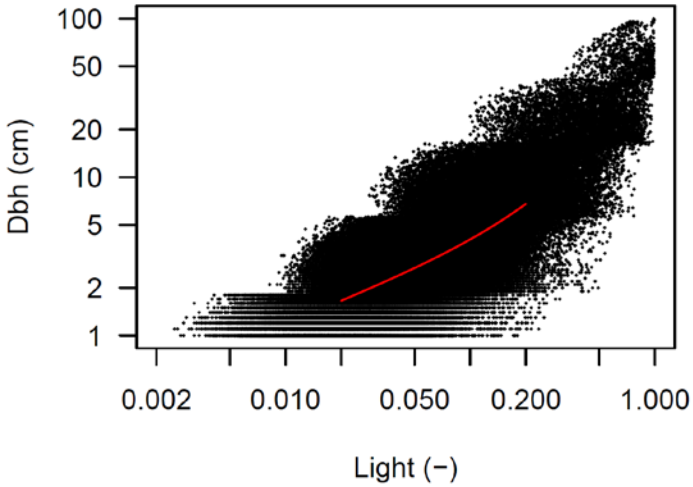

Nonlinear regression predicting average log(dbh) in the light range from 2 to 20% (log(dbh) = 4.547 + 0.455 log(light) + 2.006 light; dbh is in mm). Average log(dbh) is used to estimate average growth across the light range for Fig. 4 of the main manuscript.
