# Supplementary figures and images for: Growth Strategies of Tropical Tree Species: Disentangling Light and Size Effects
Source: PLoS One. 2011 Sep 22;6(9):e25330. doi: 10.1371/journal.pone.0025330 (PMC3178650; doi:10.1371/journal.pone.0025330)

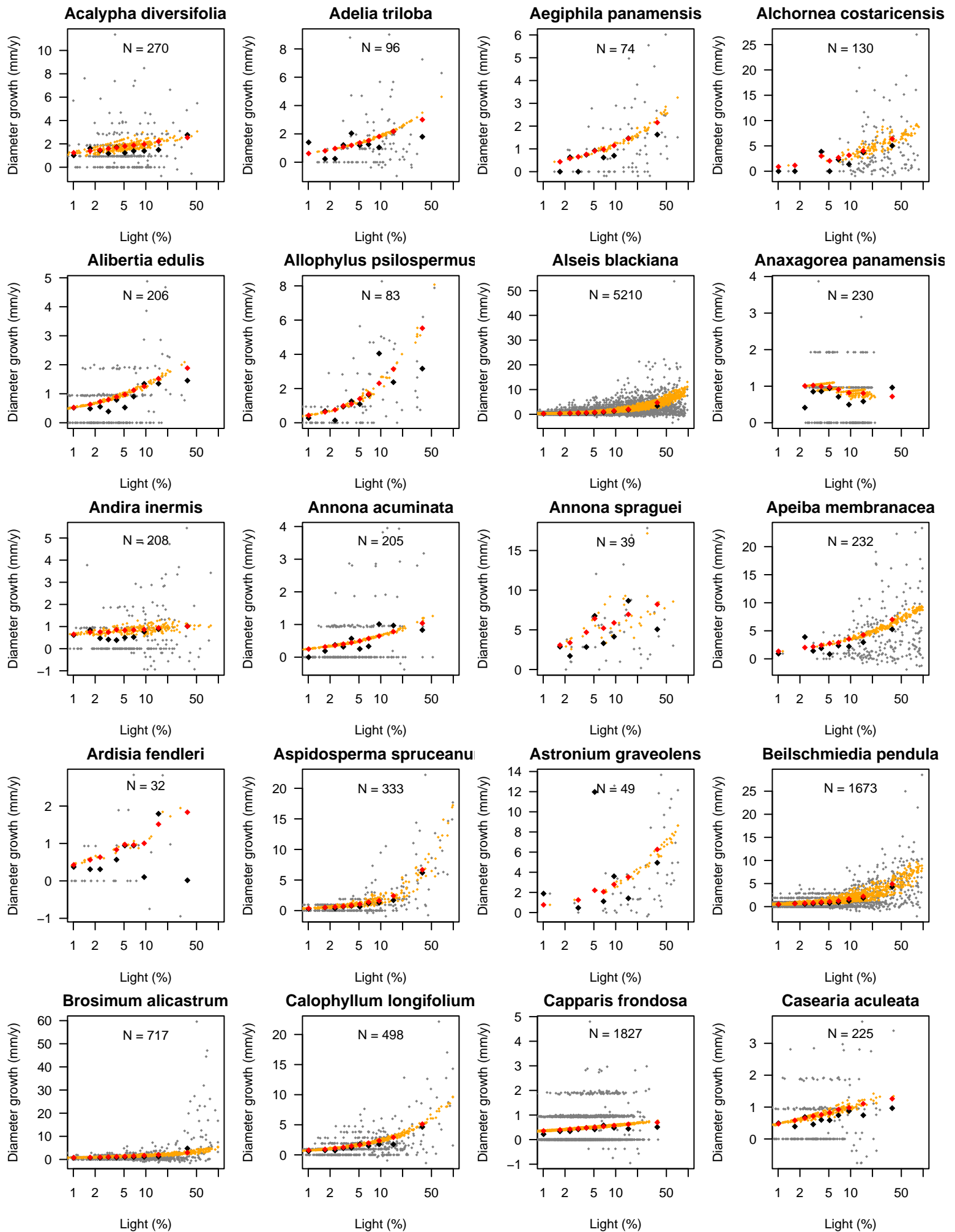

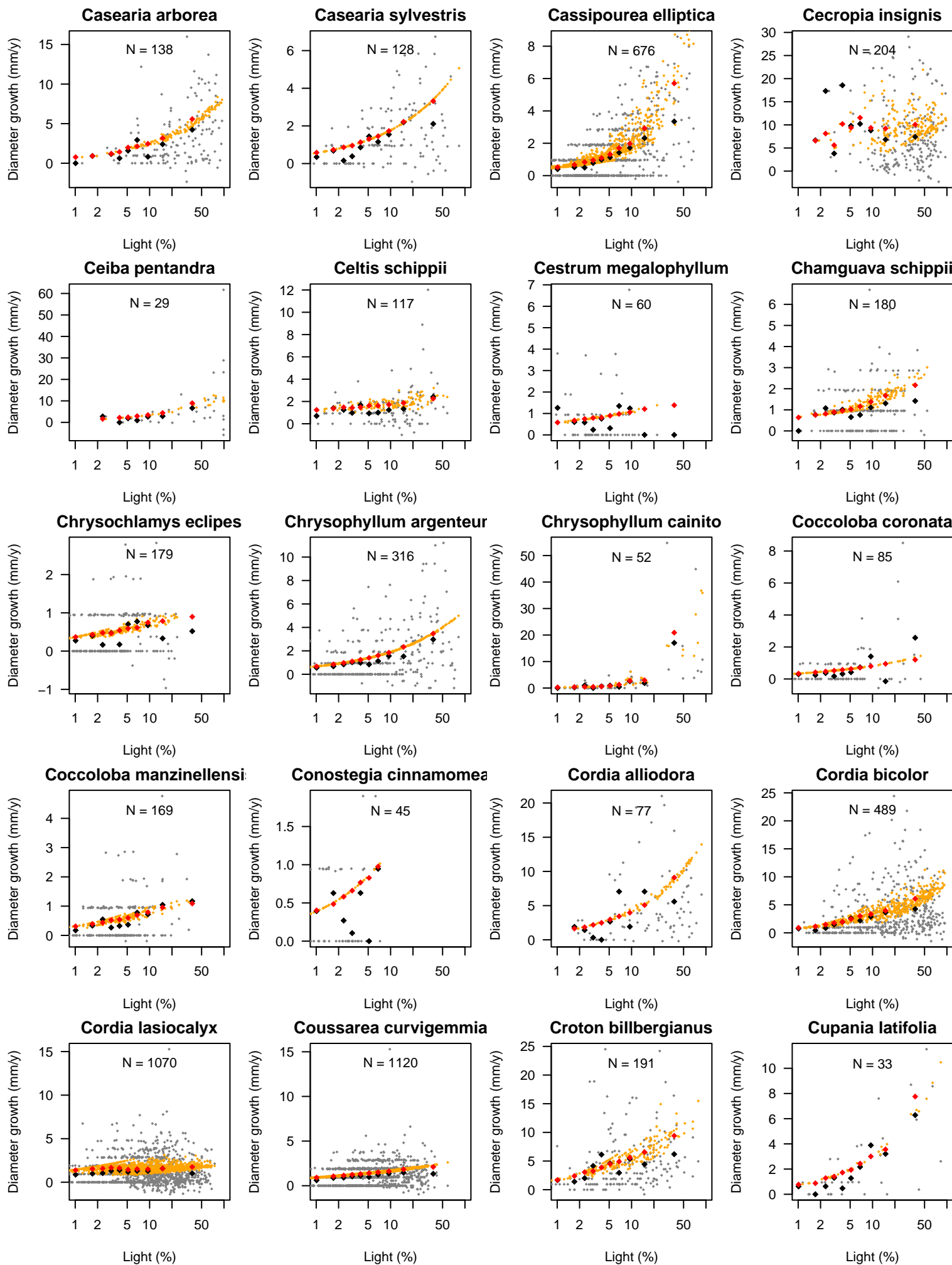

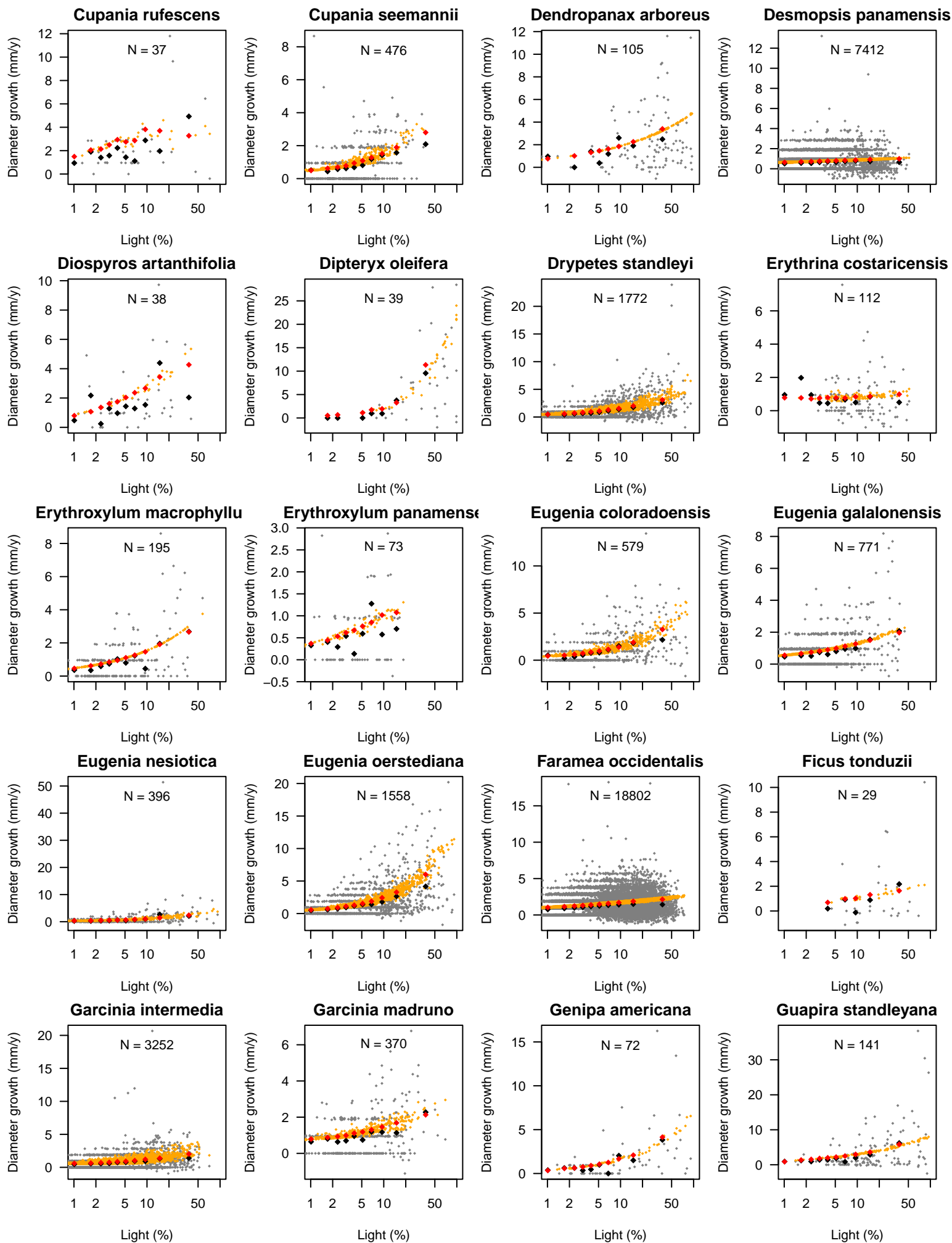

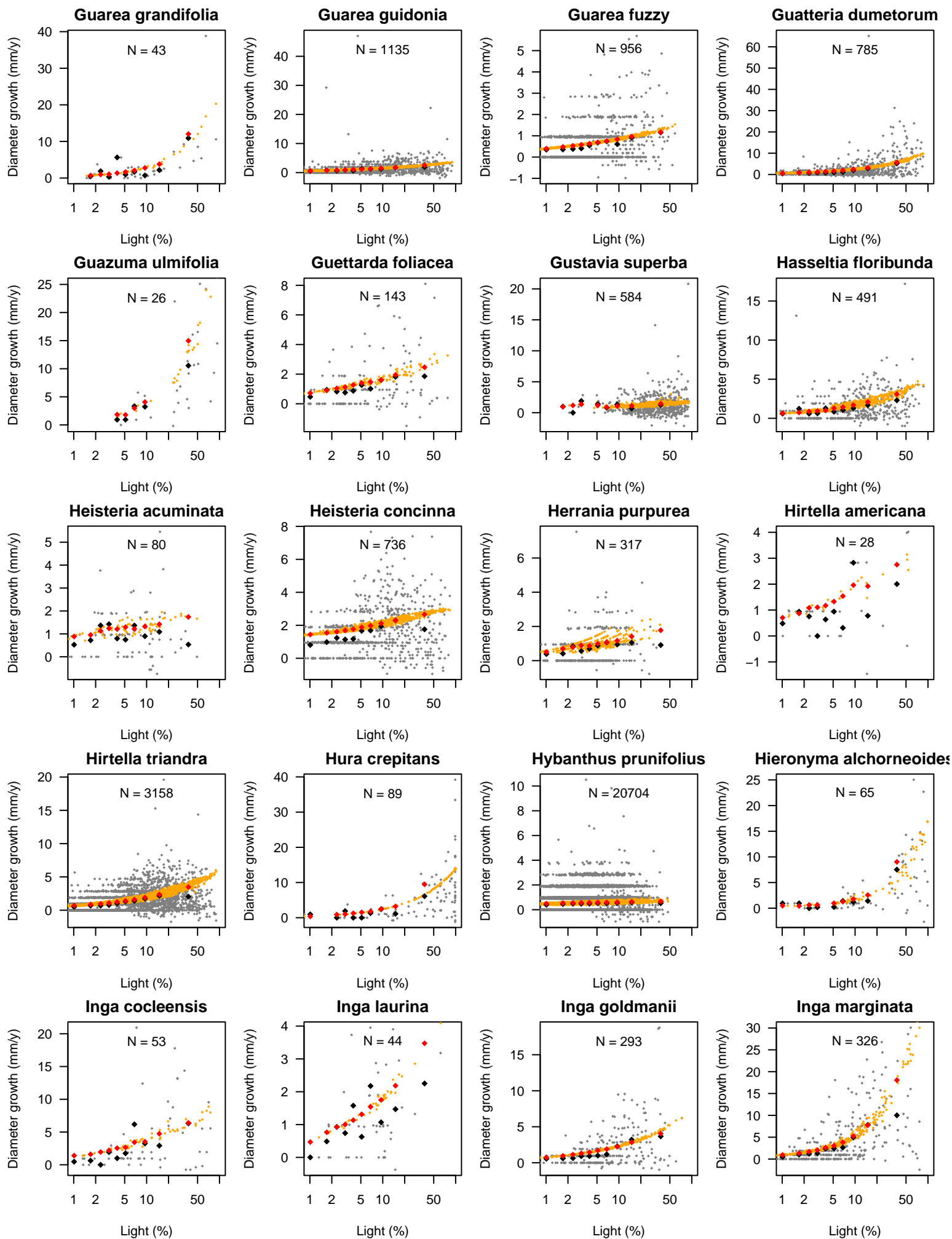

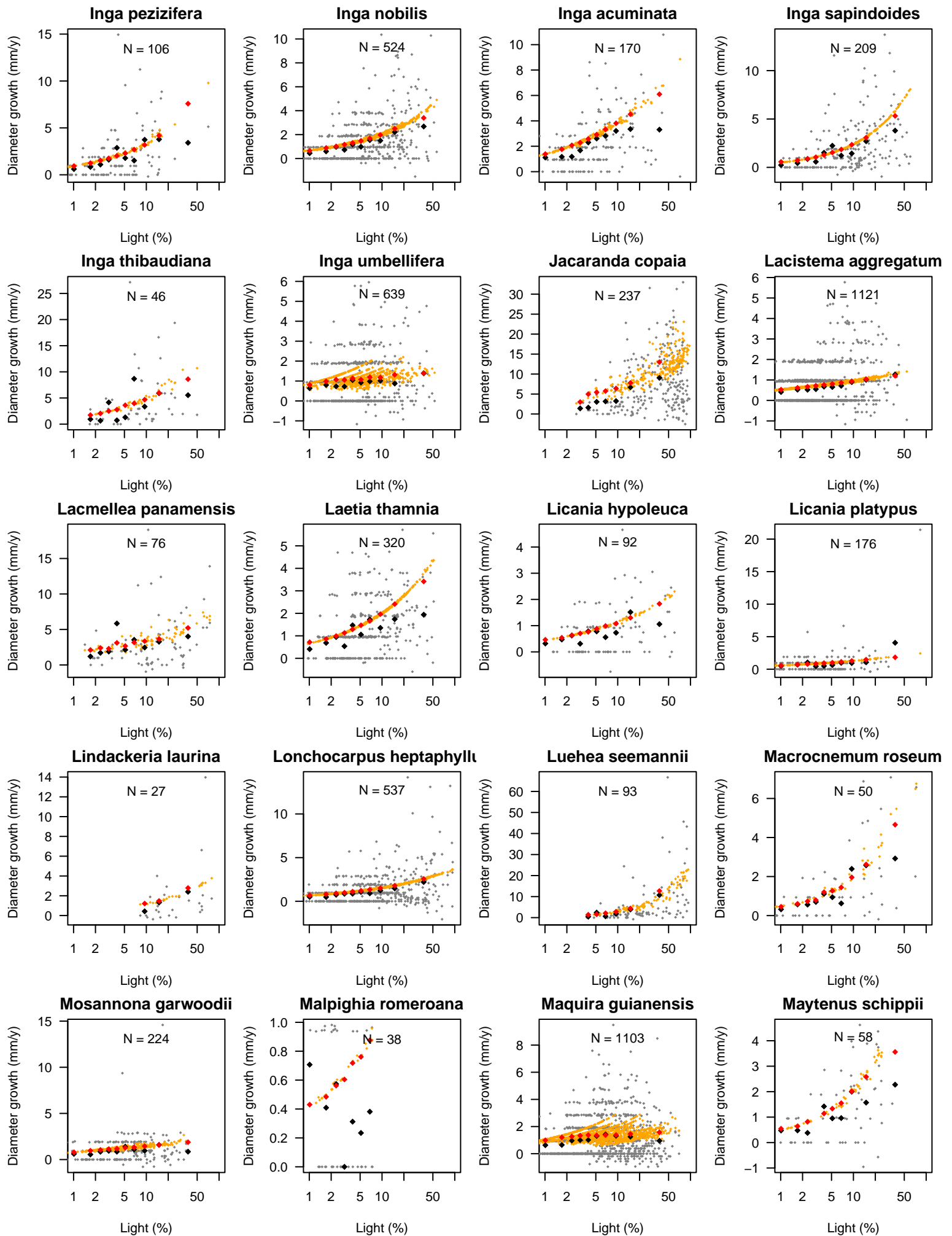

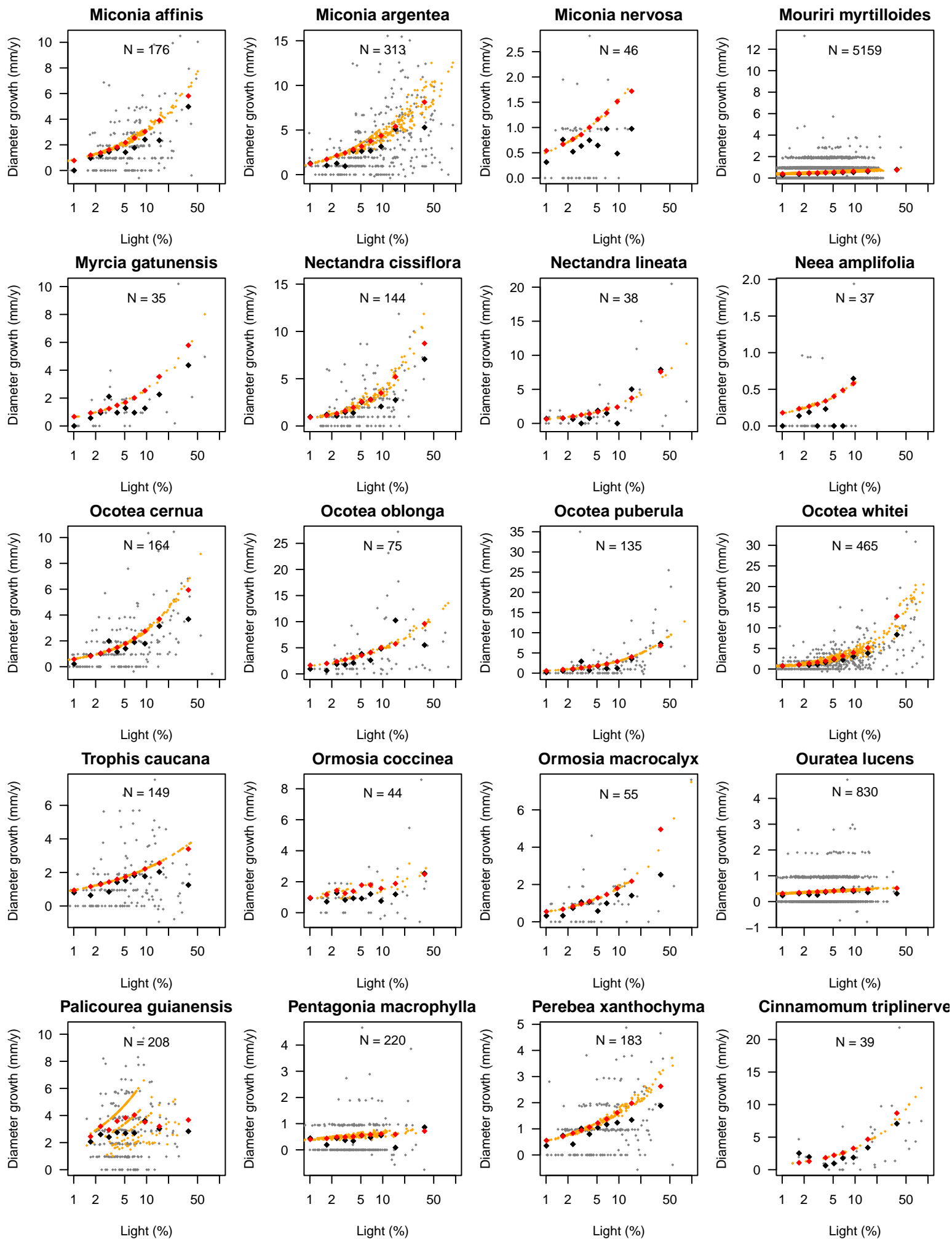

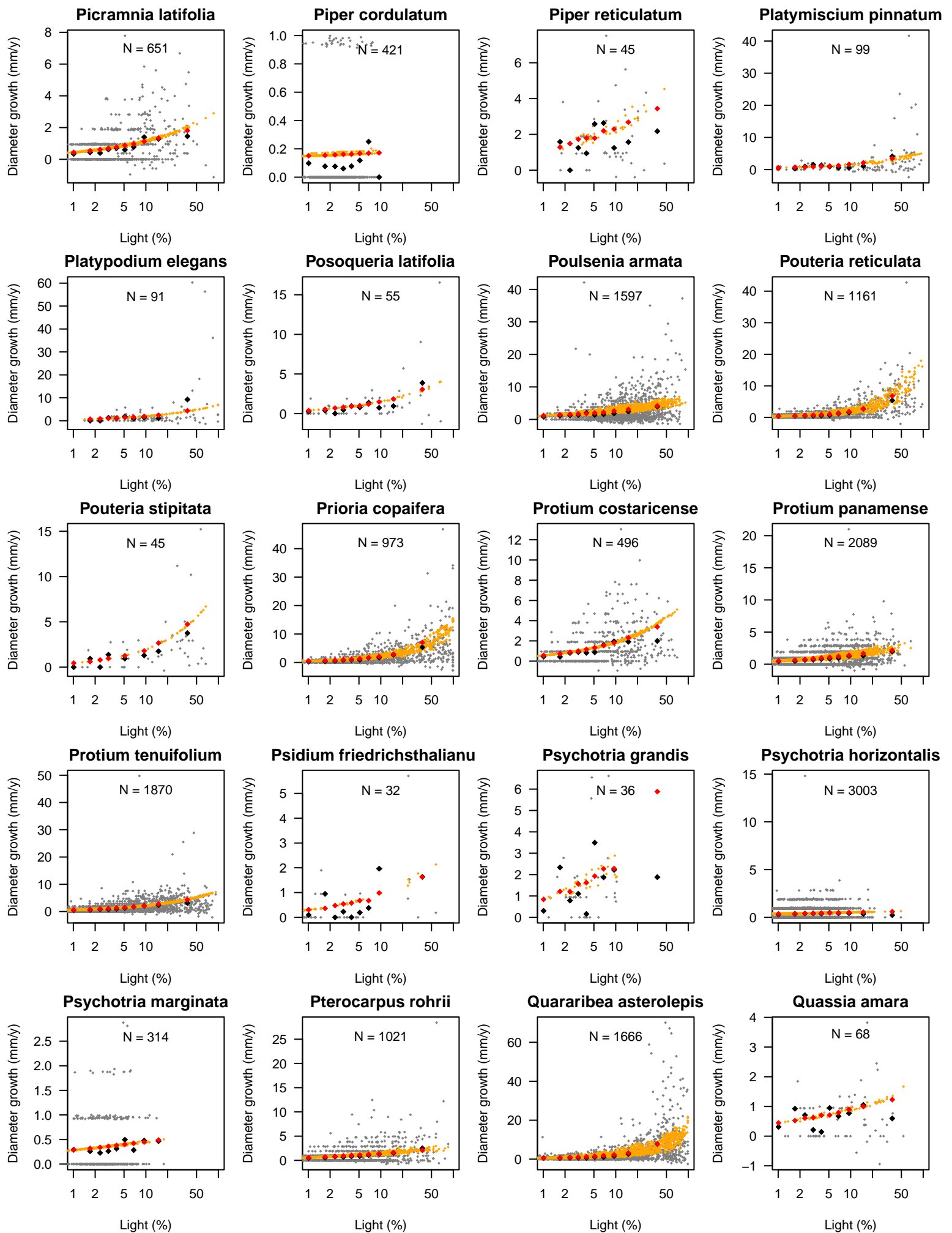

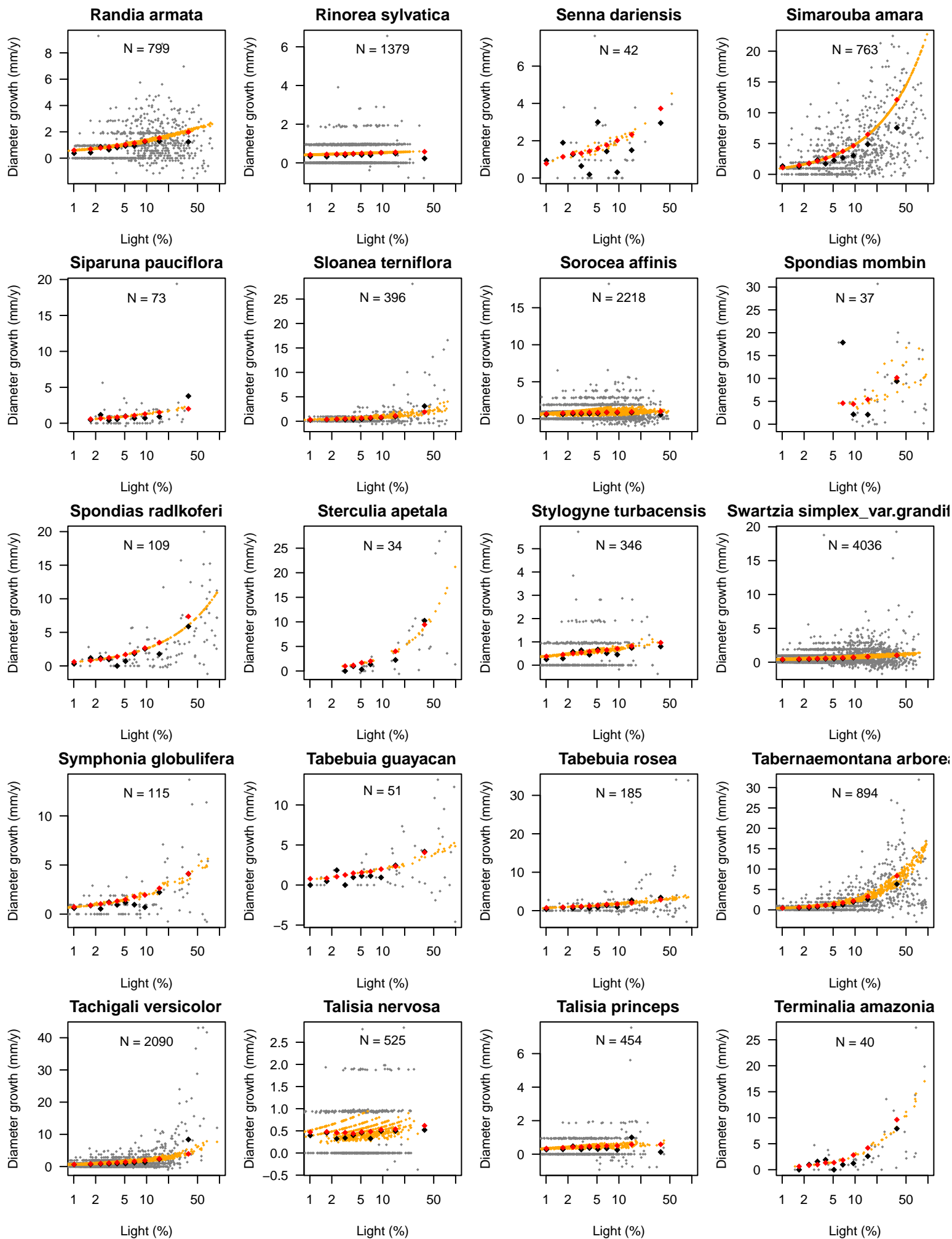

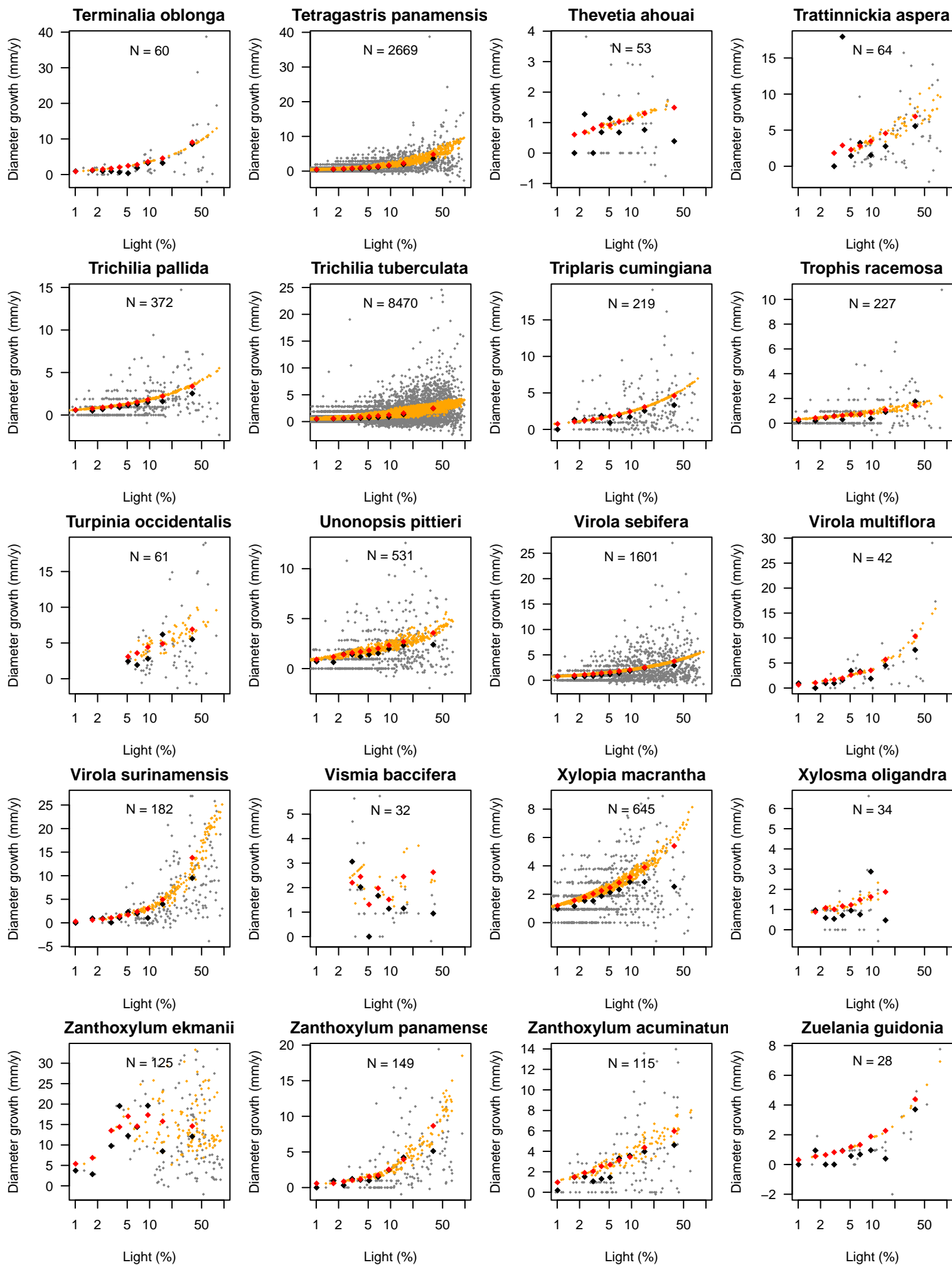

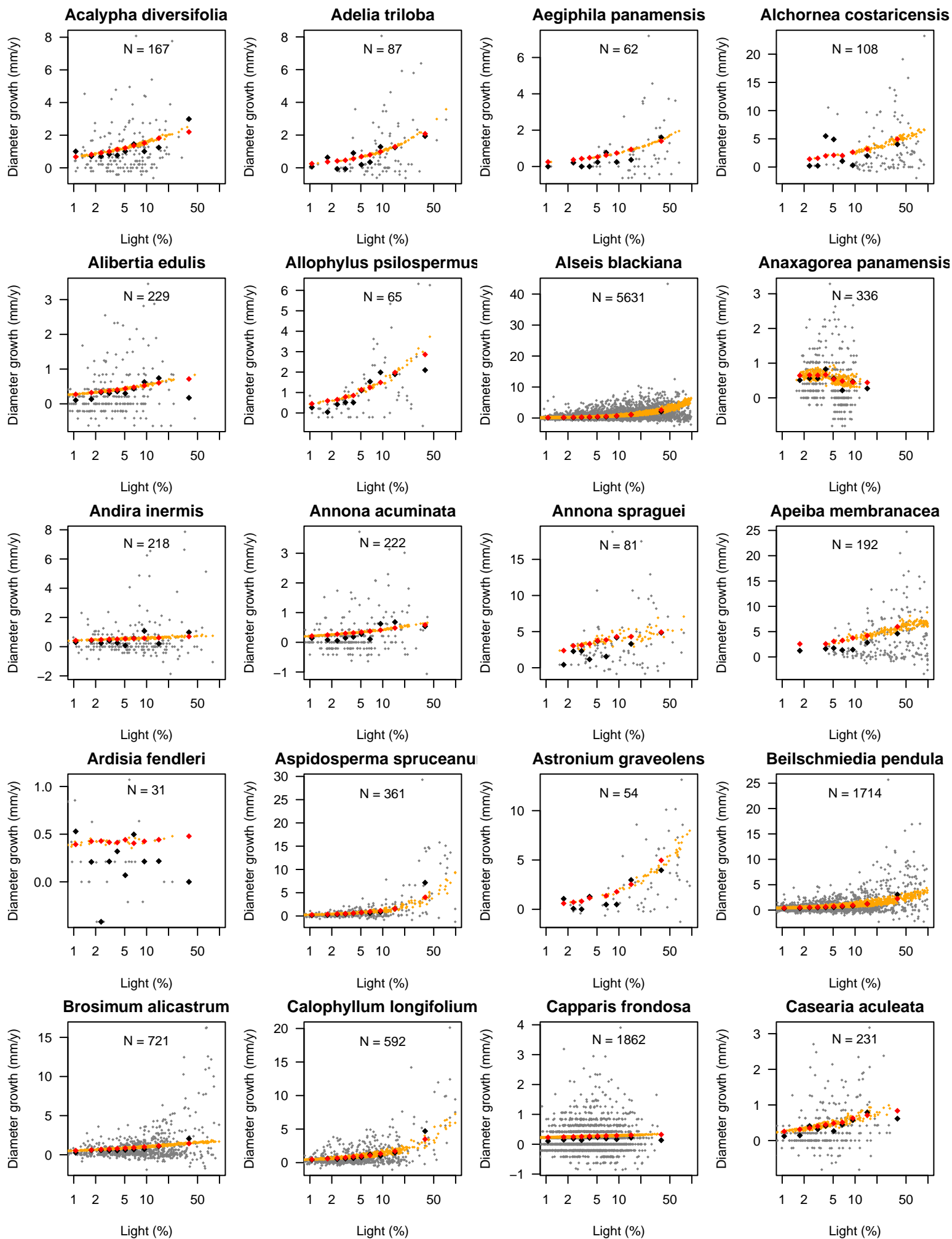

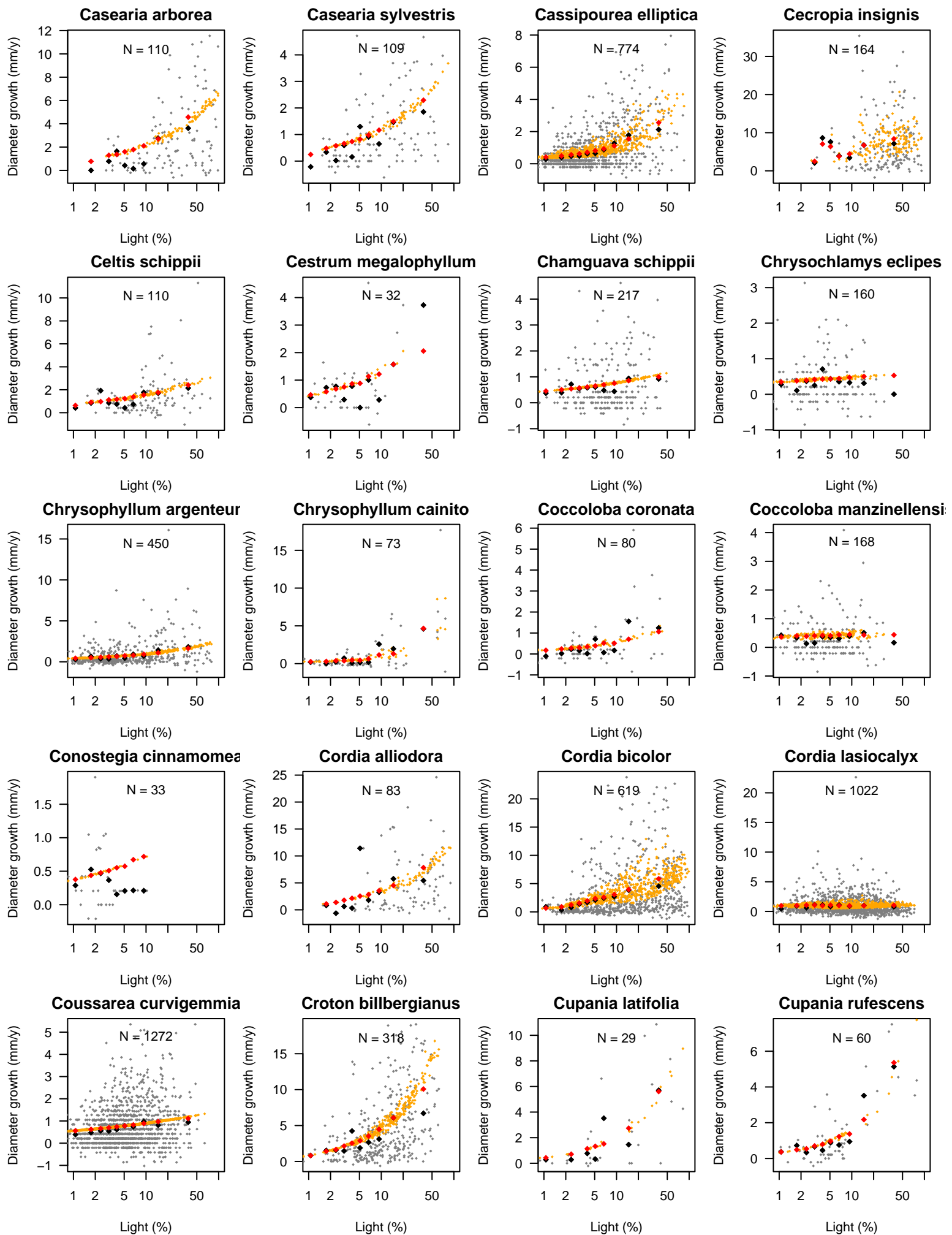

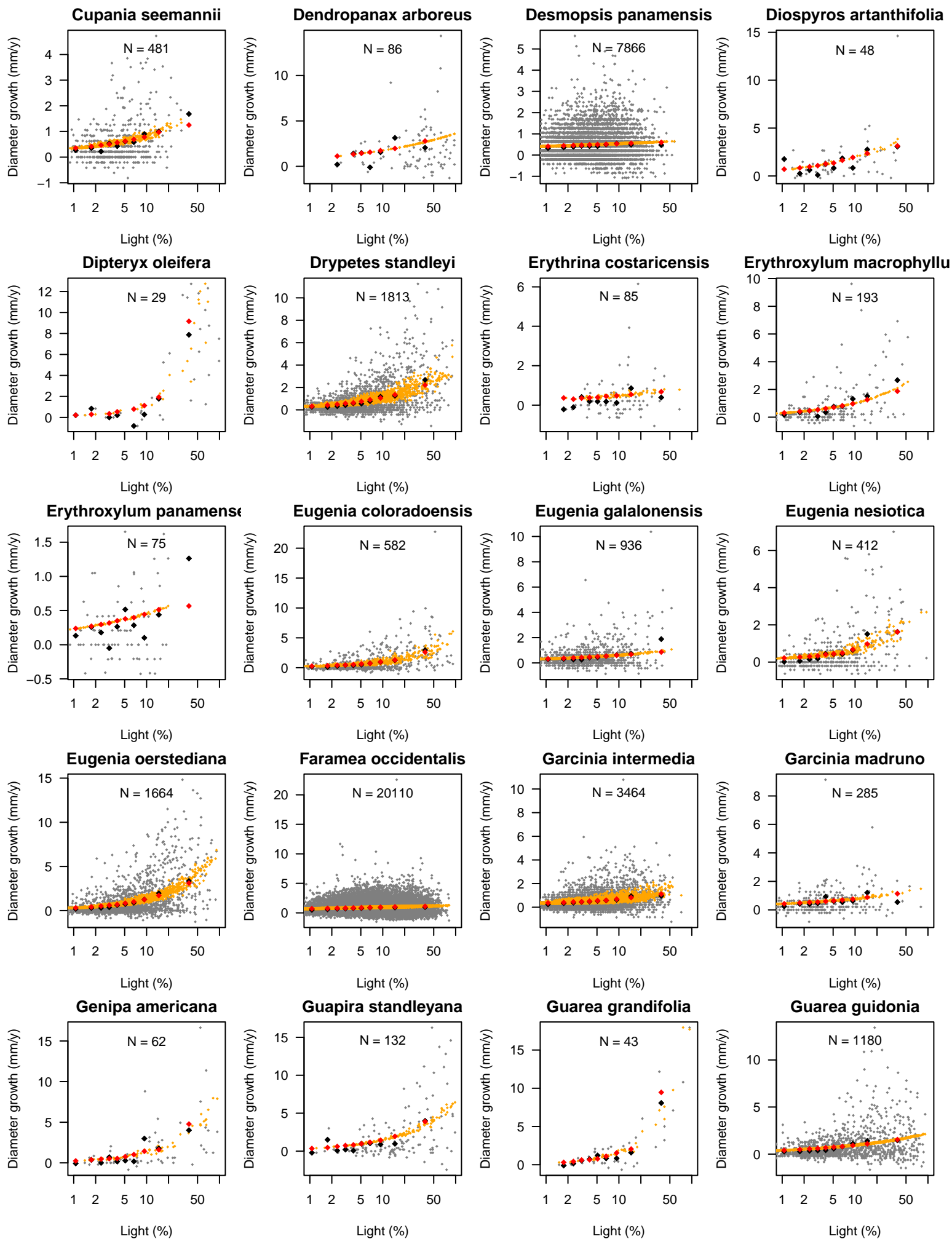

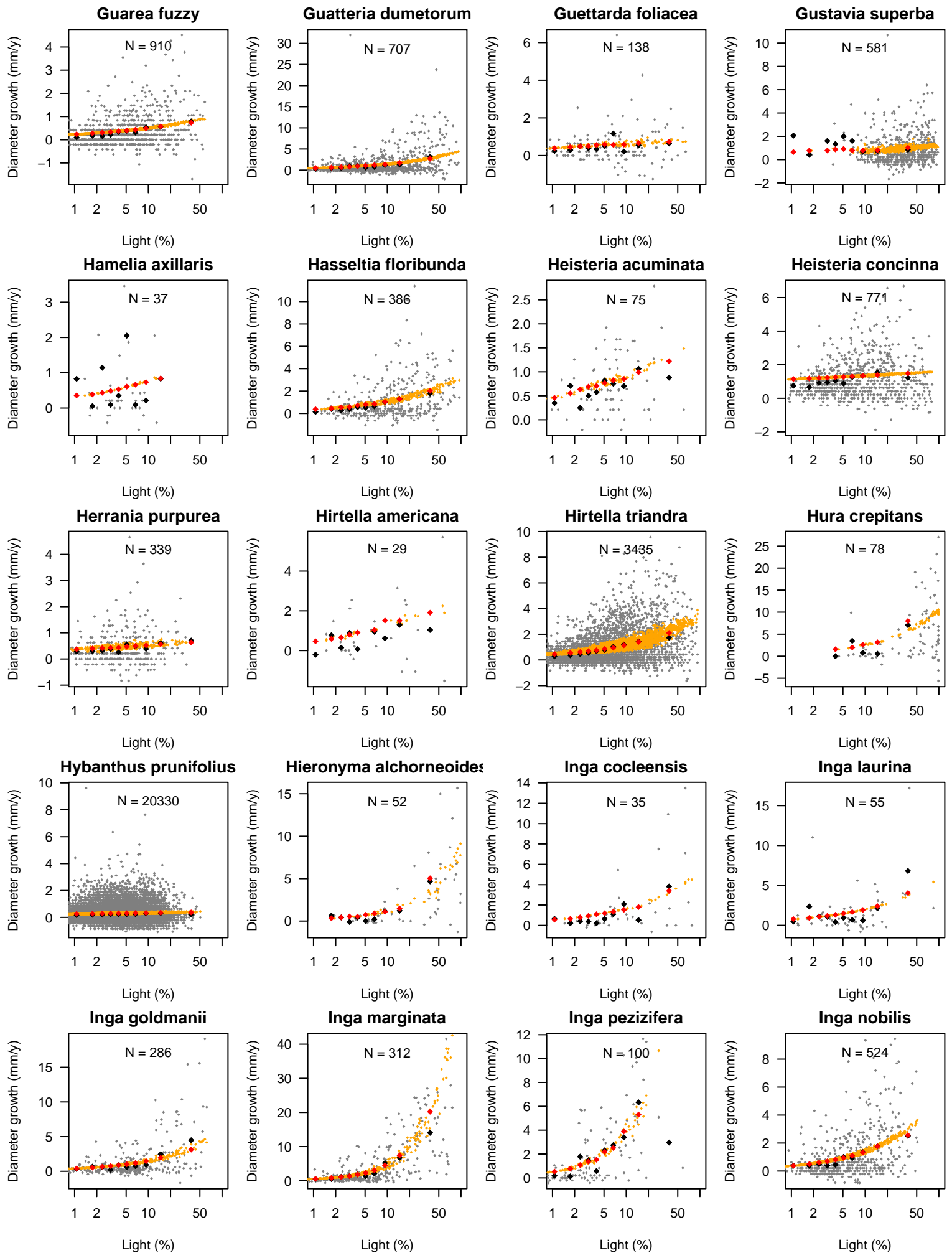

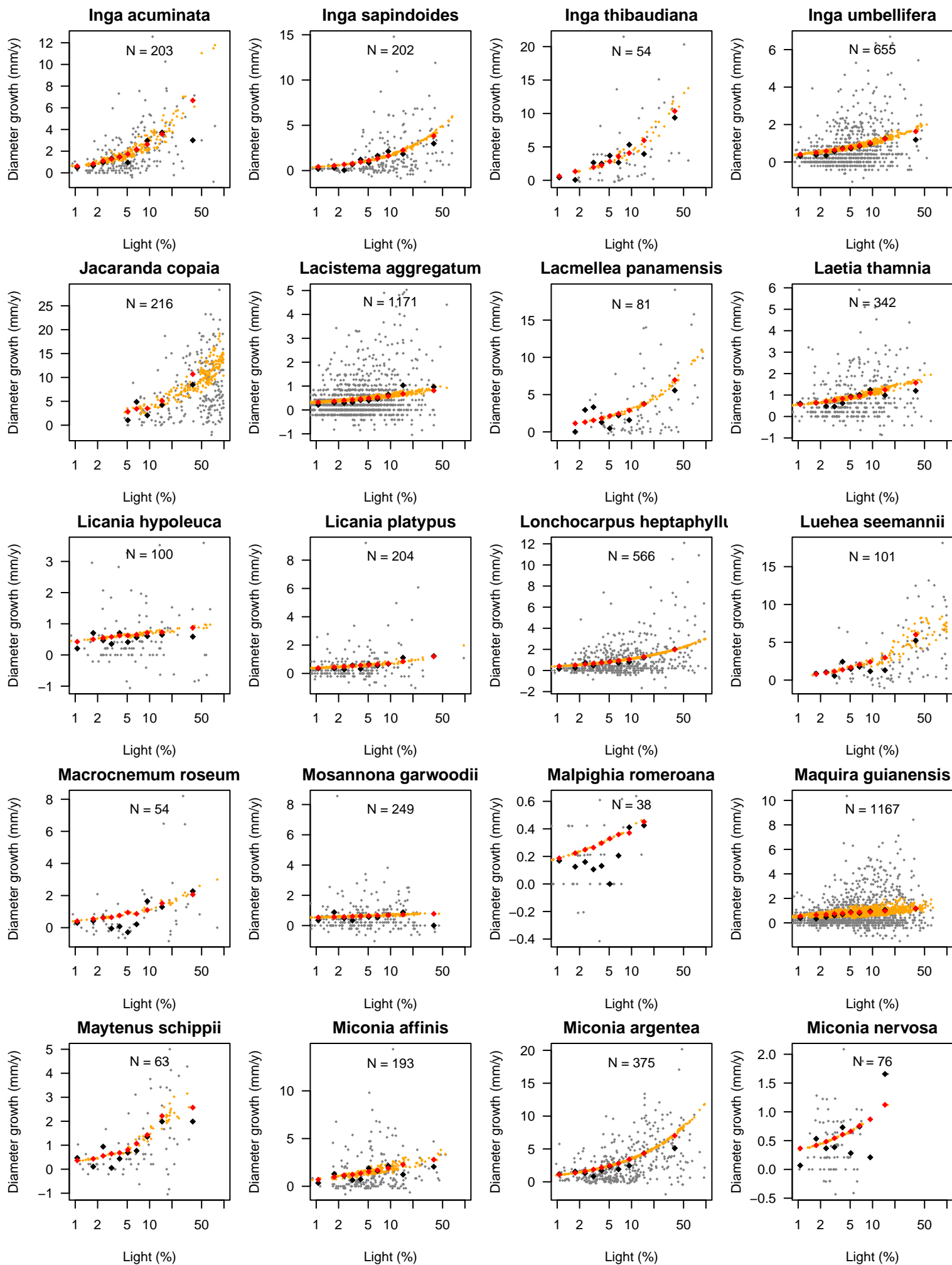

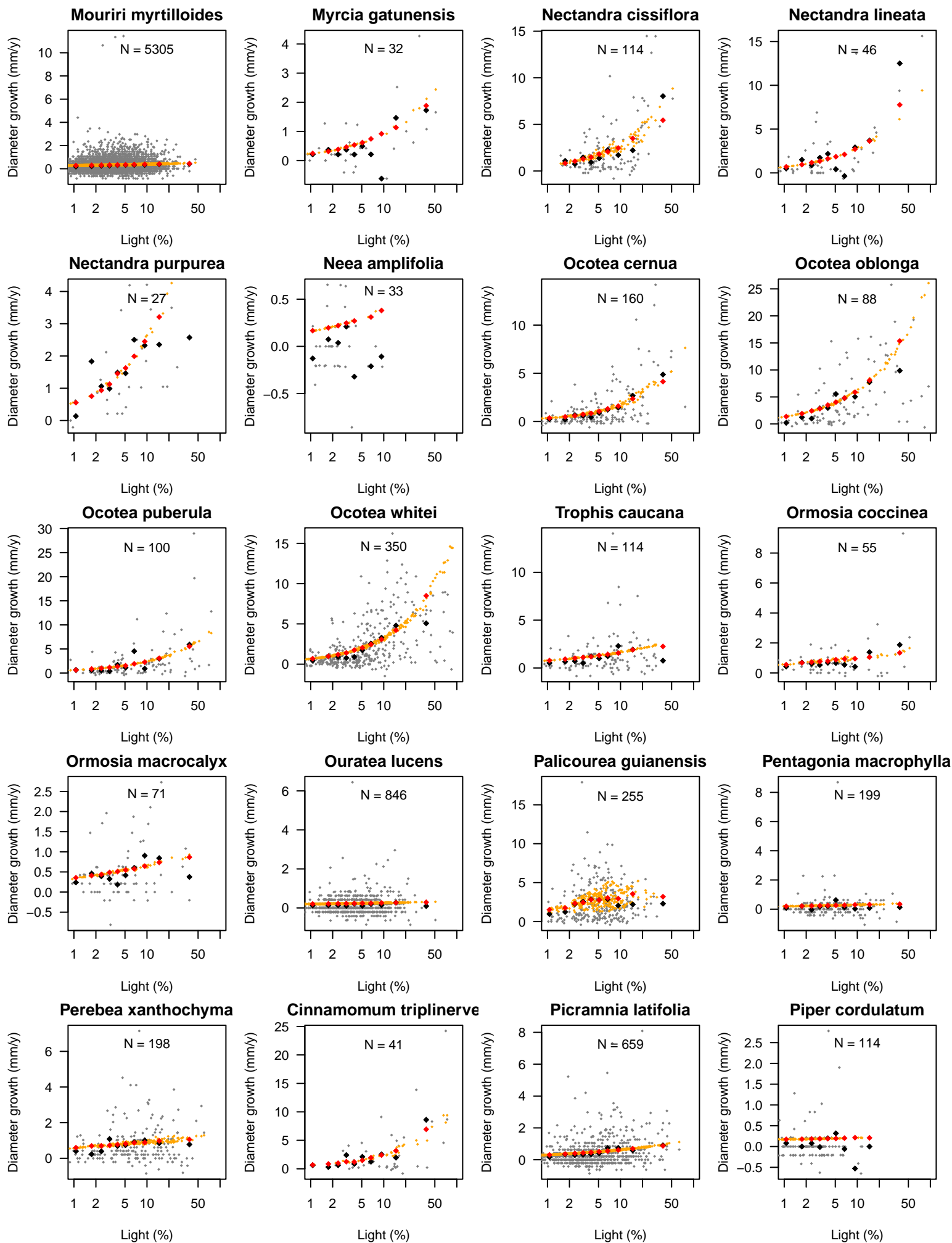

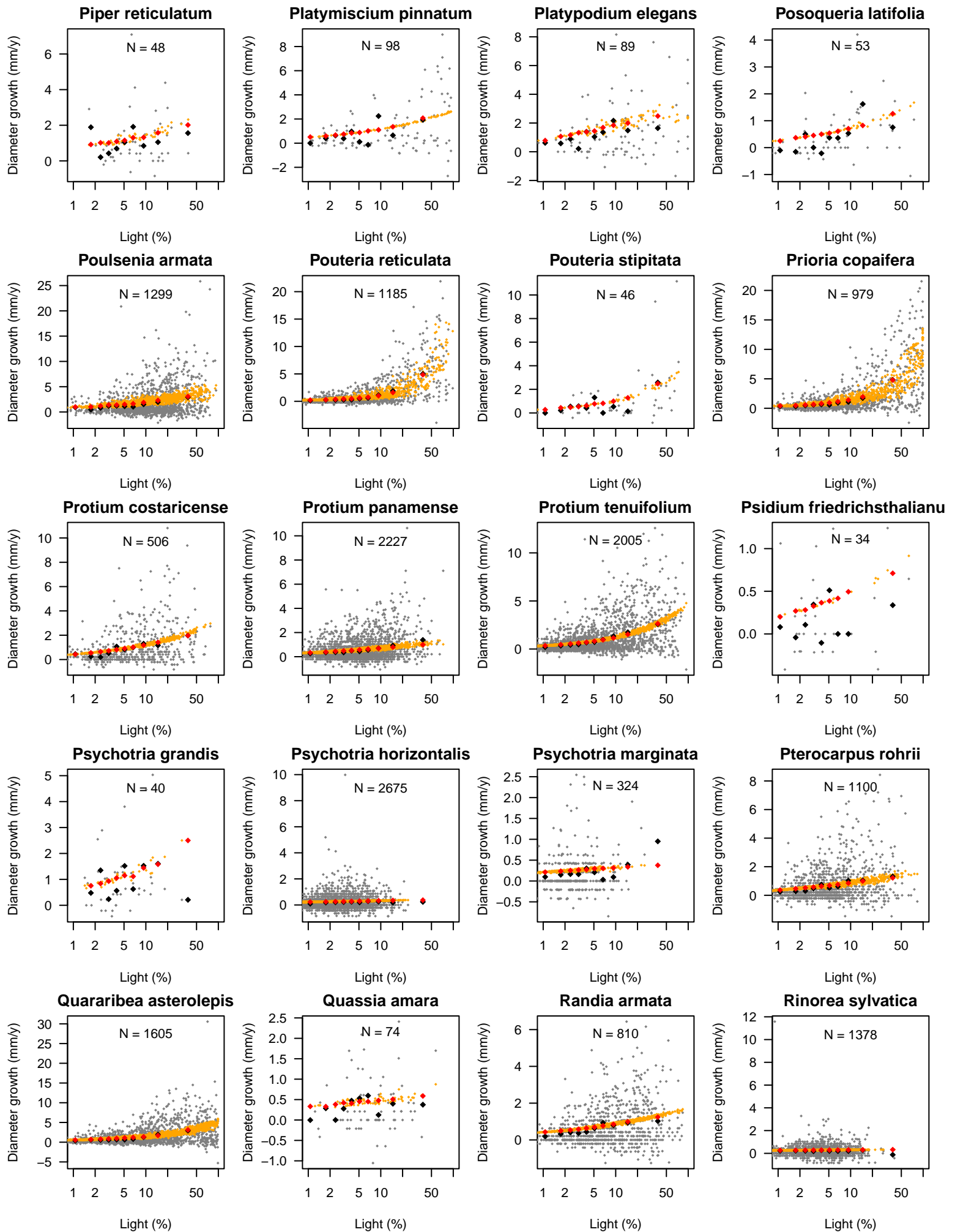

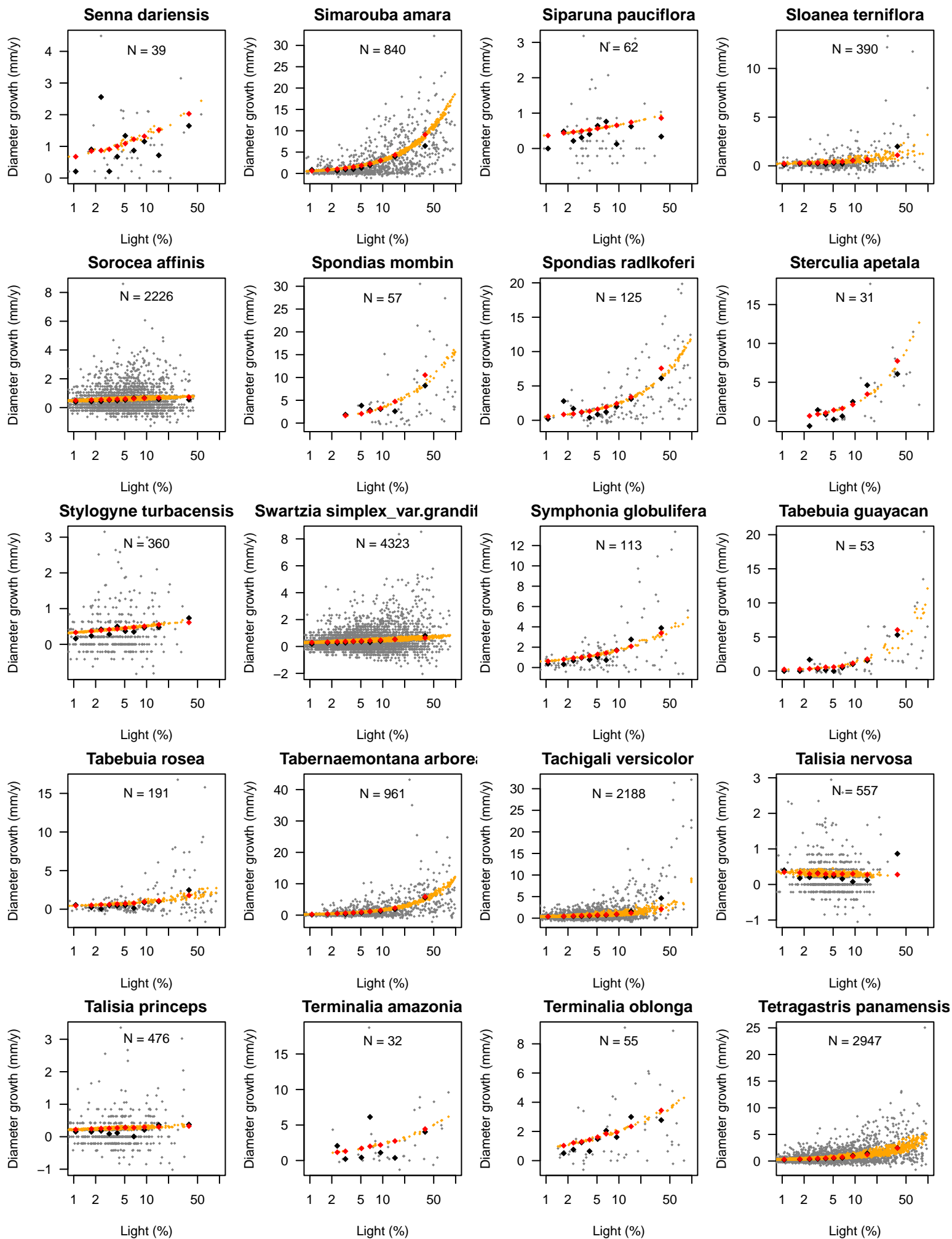

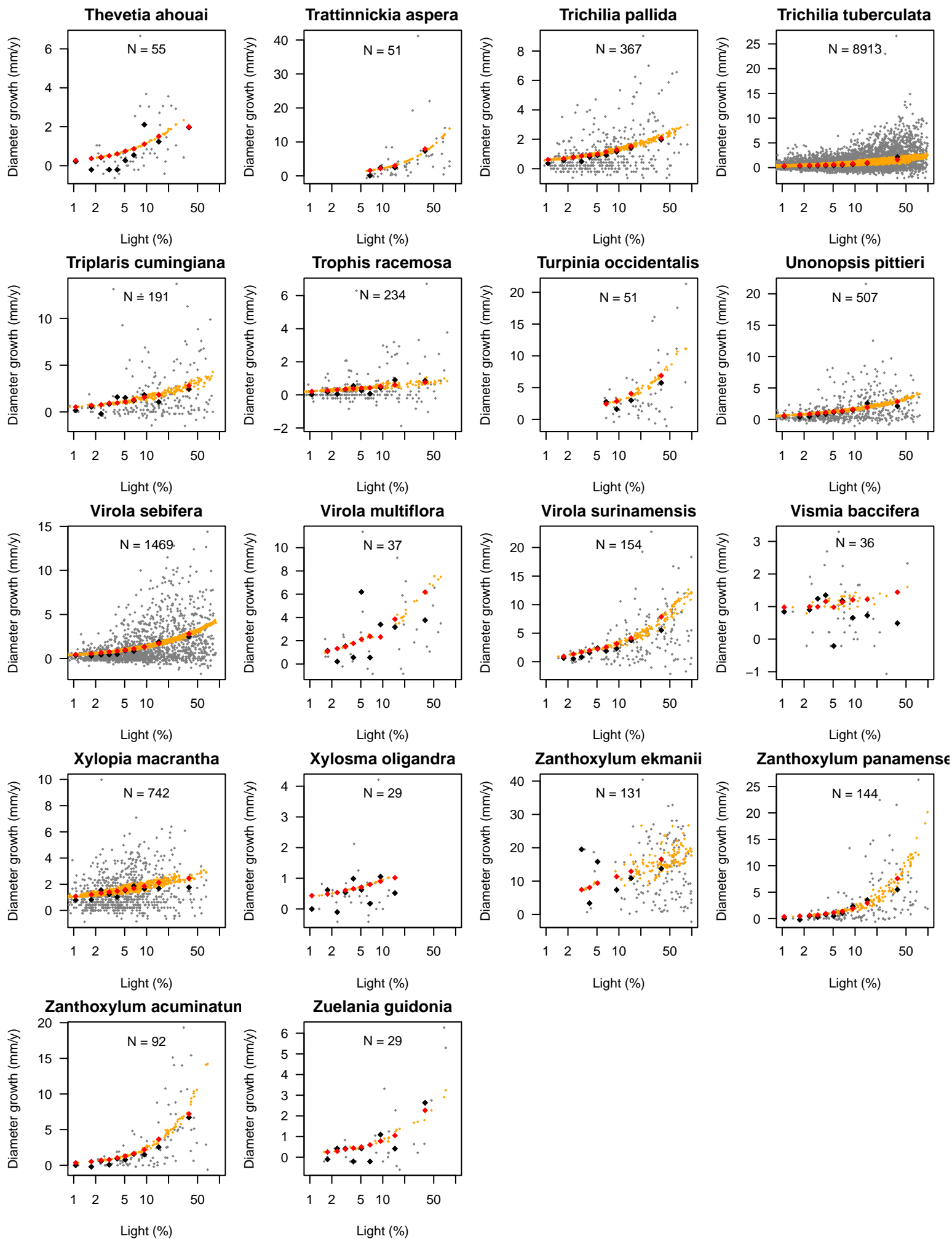

Supplement: Figure S2 — Light dependence of growth rates for species with ≥25 individuals in the two census intervals (1985−1990, 1990−1995). Light classes correspond to deciles of light availability across all individuals. Predicted mean growth rates were calculated at mean observed light level and mean dbh of the individuals in the respective light class. Observed and predicted growth rates of individual trees are displayed as grey and orange dots, respectively. Mean observed and predicted growth rates in different light classes are displayed as black and red dot, respectively. (PDF) [file pone.0025330.s002.pdf]

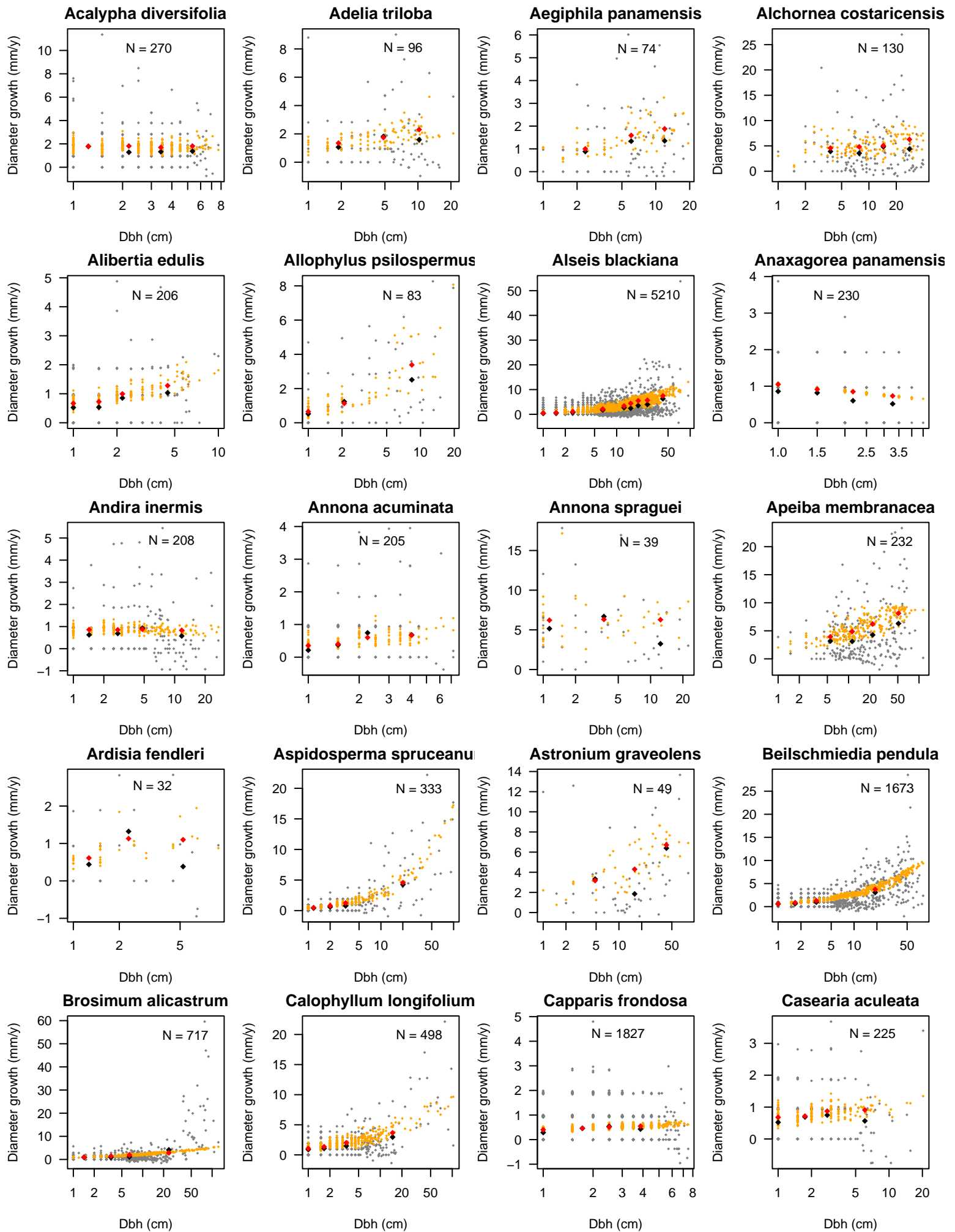

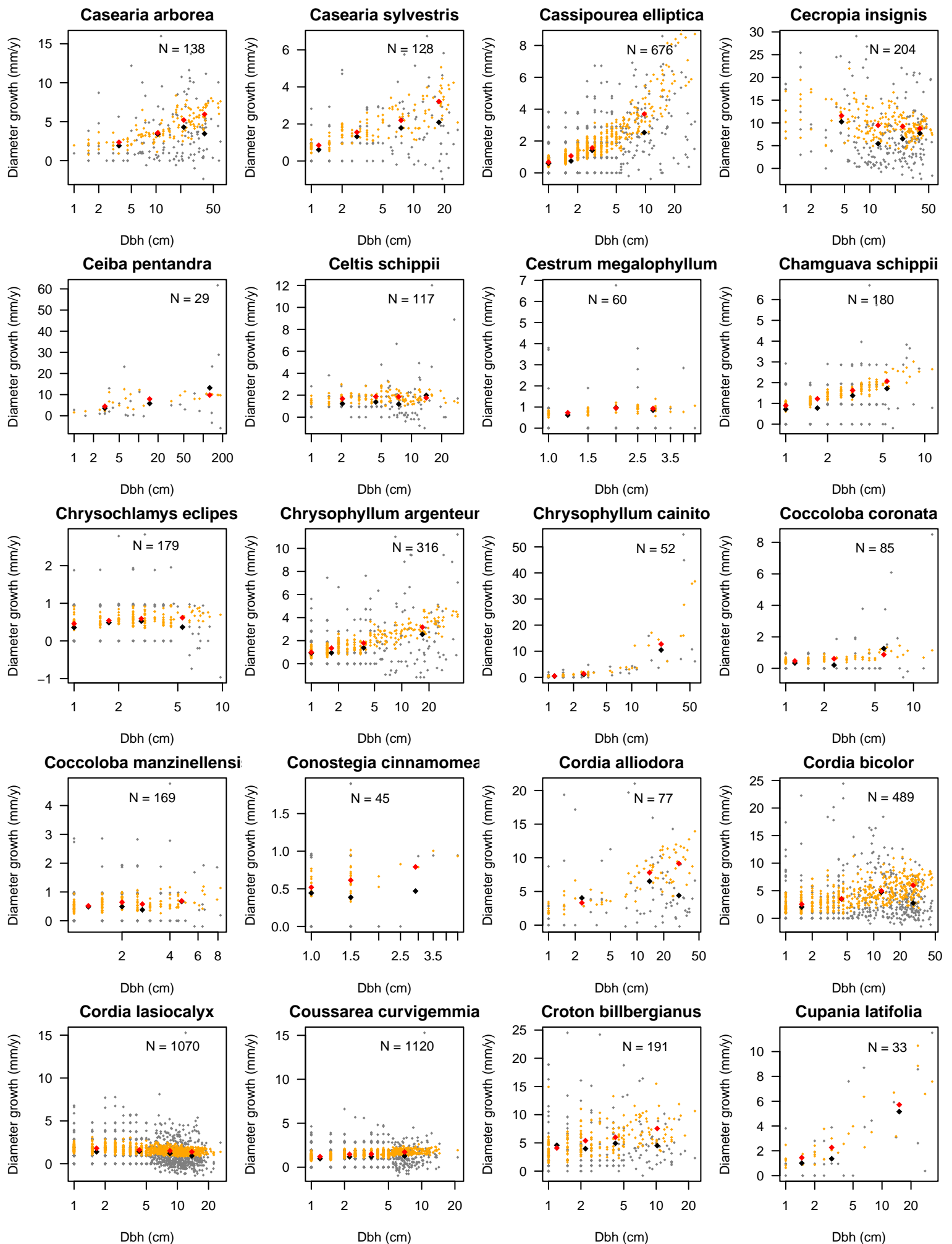

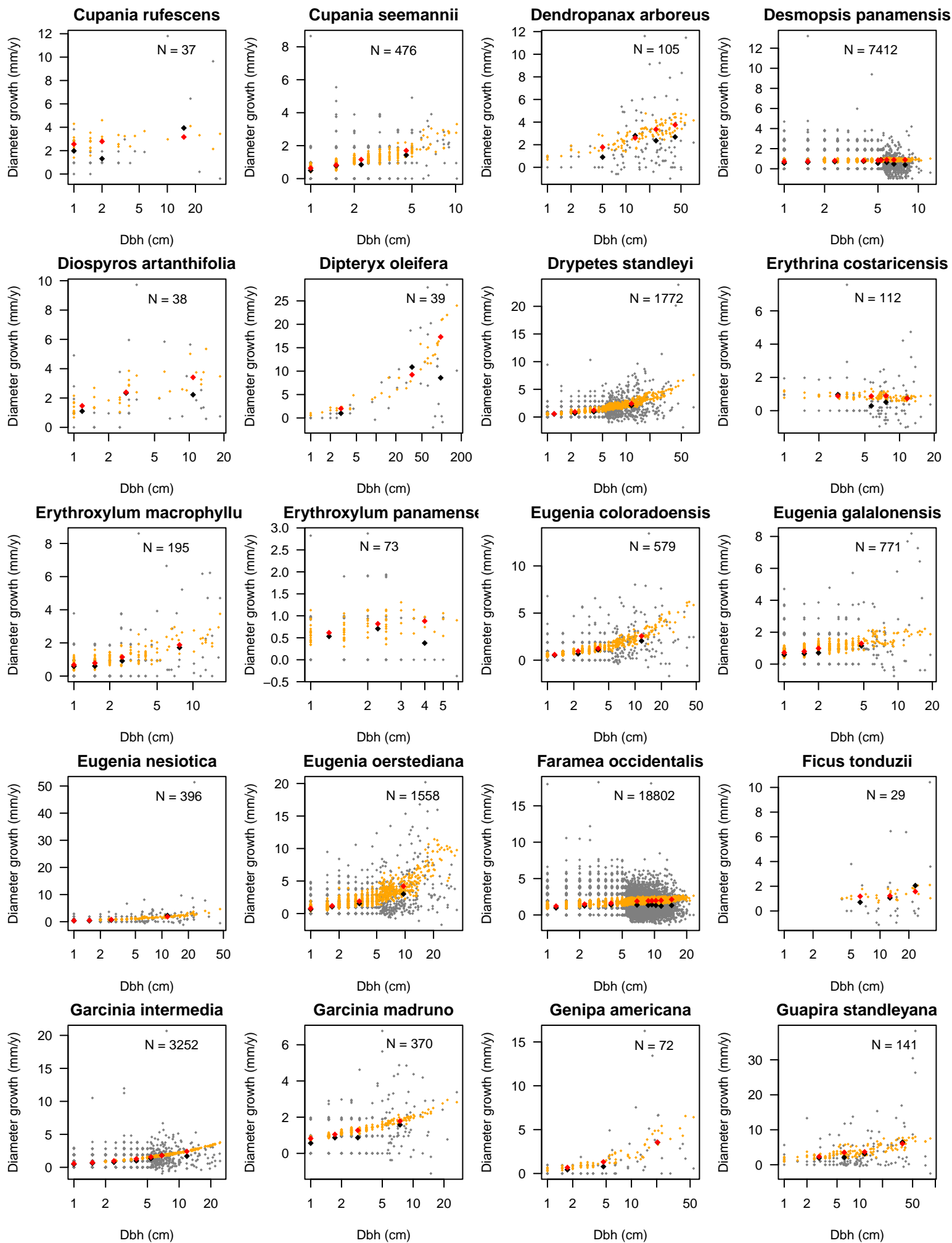

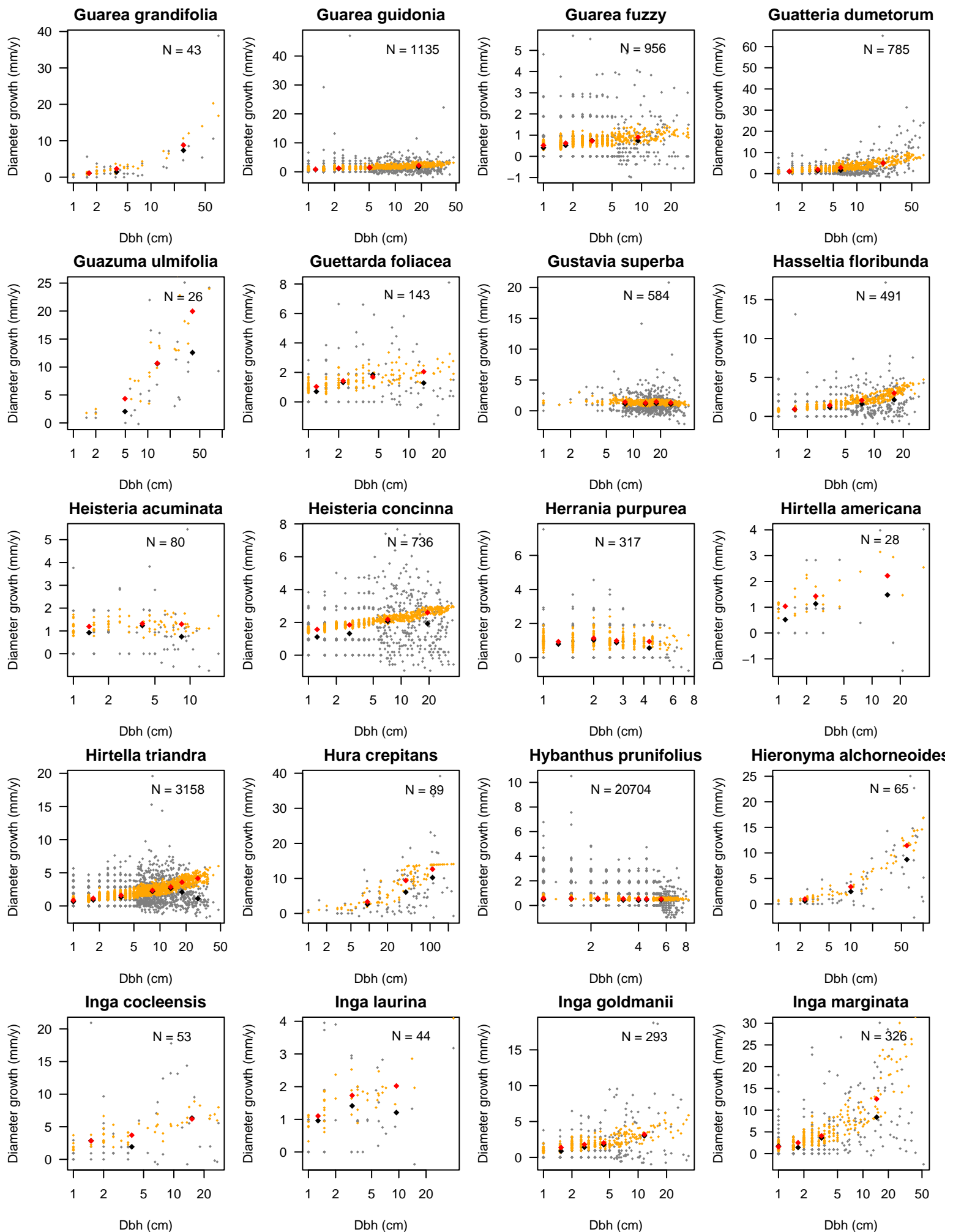

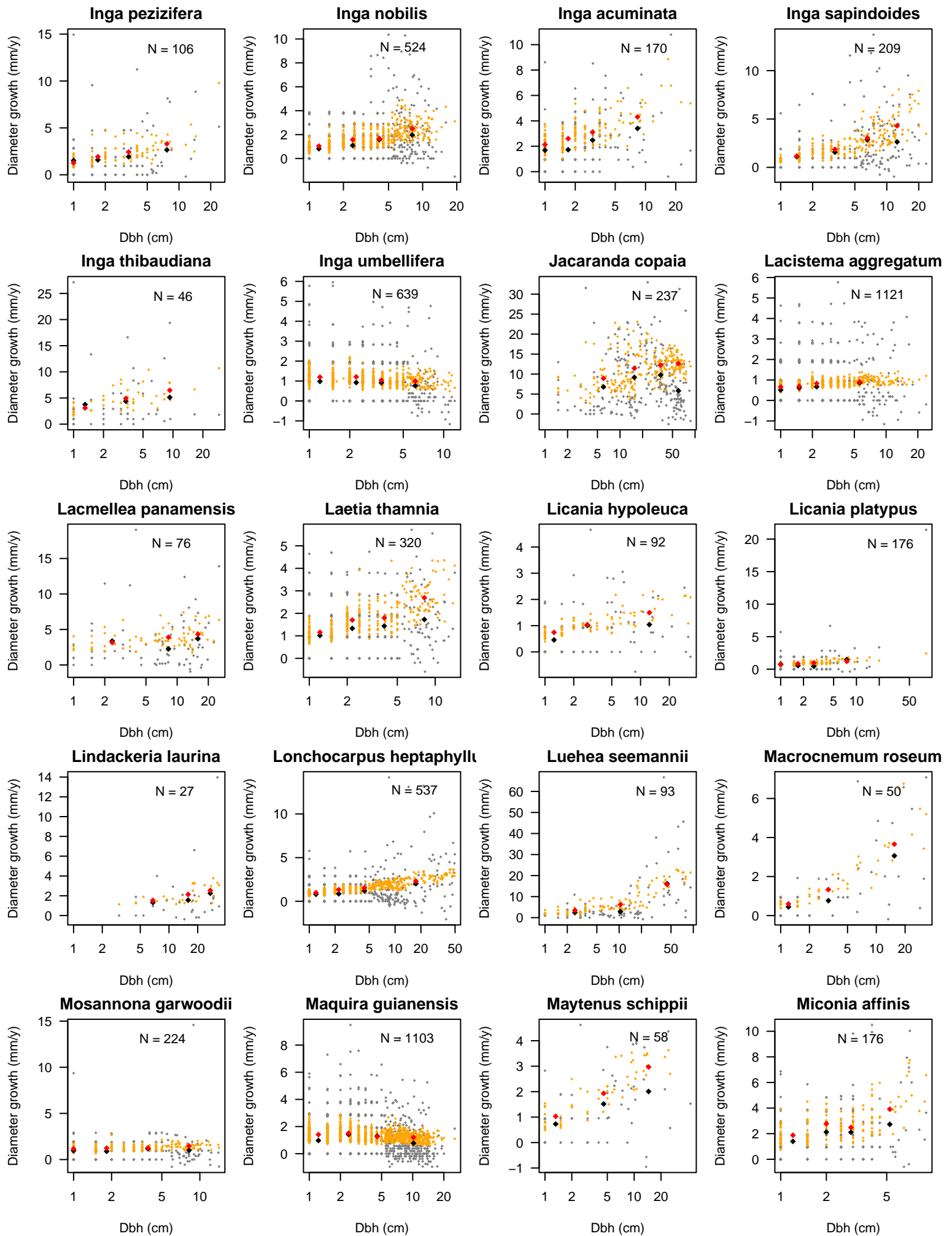

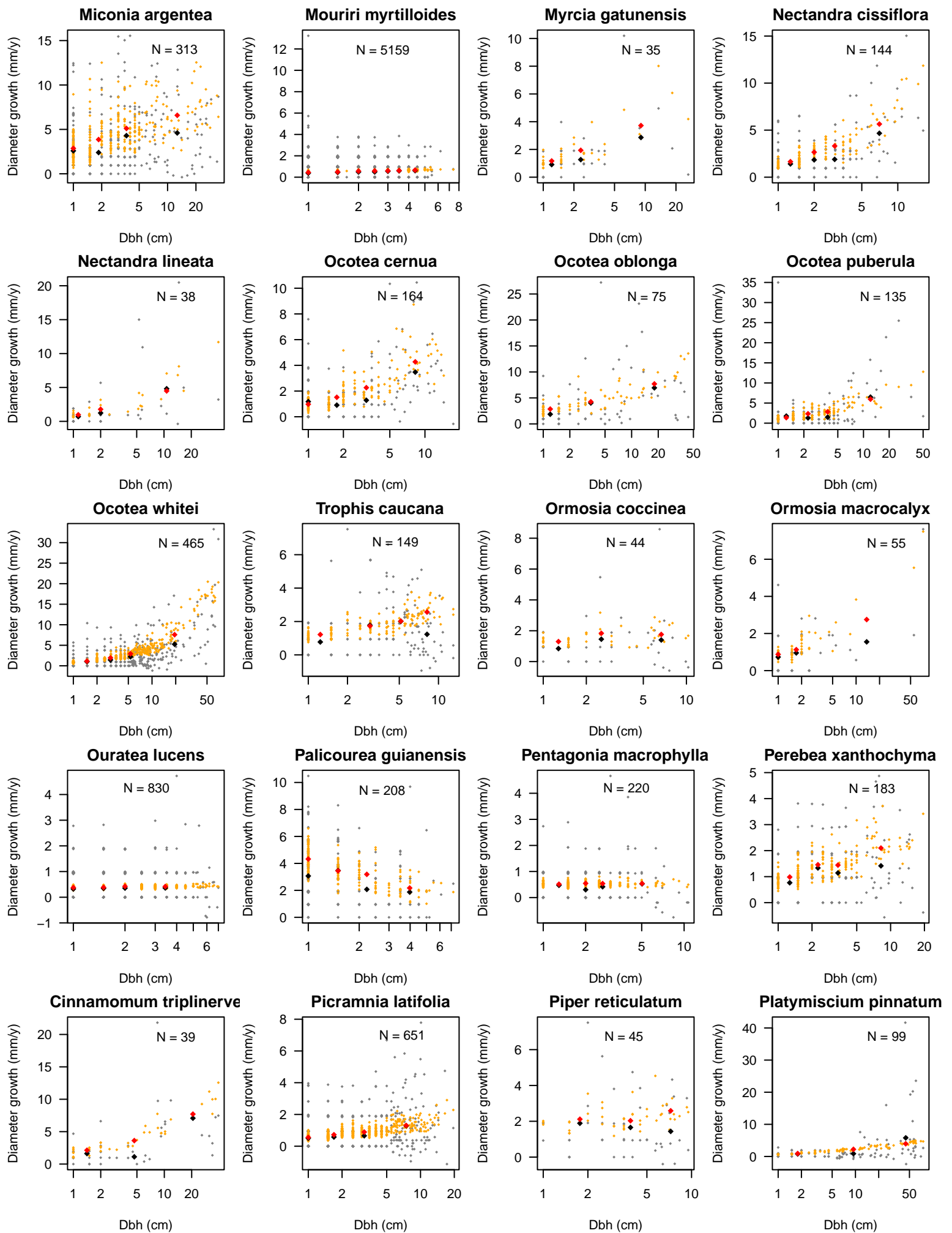

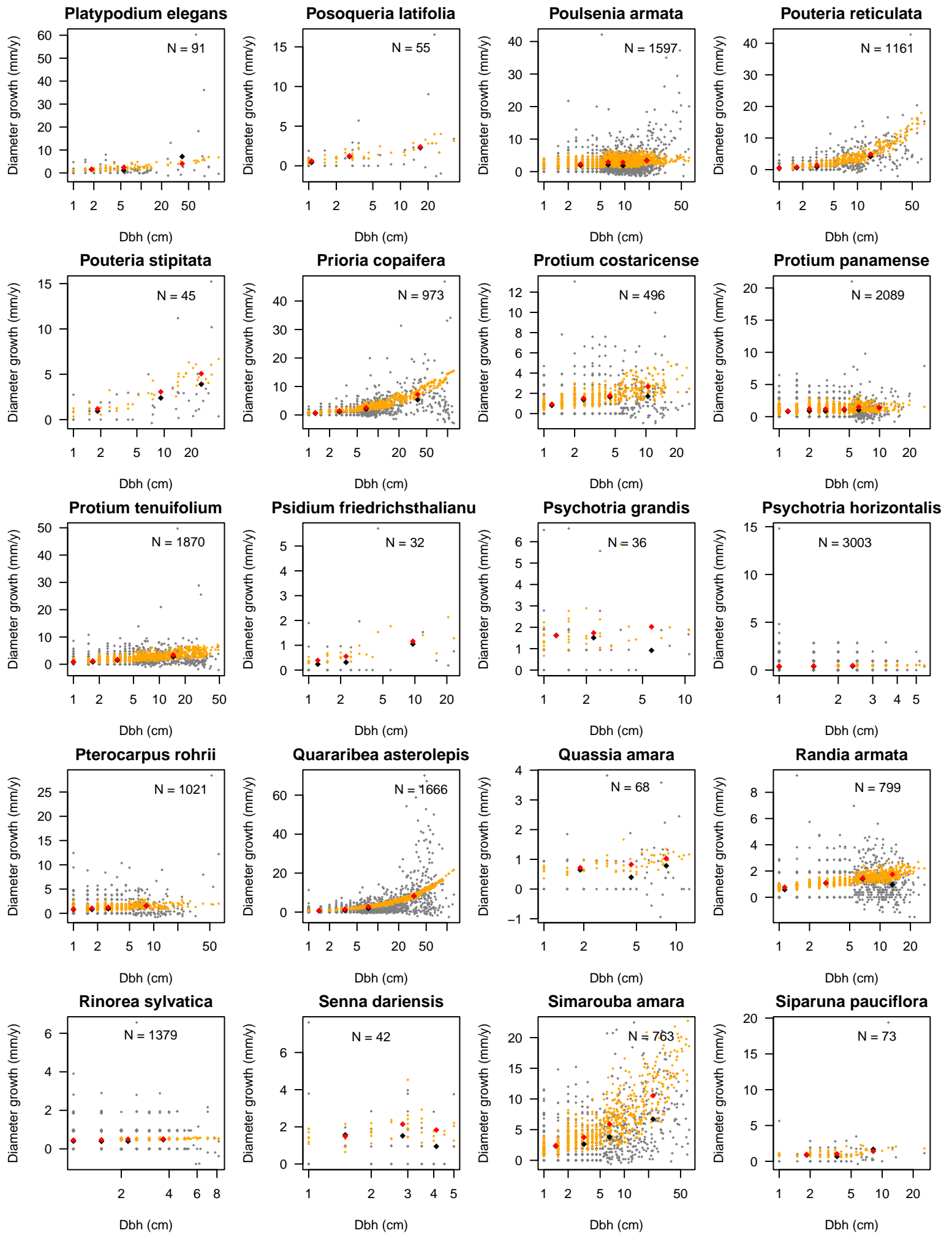

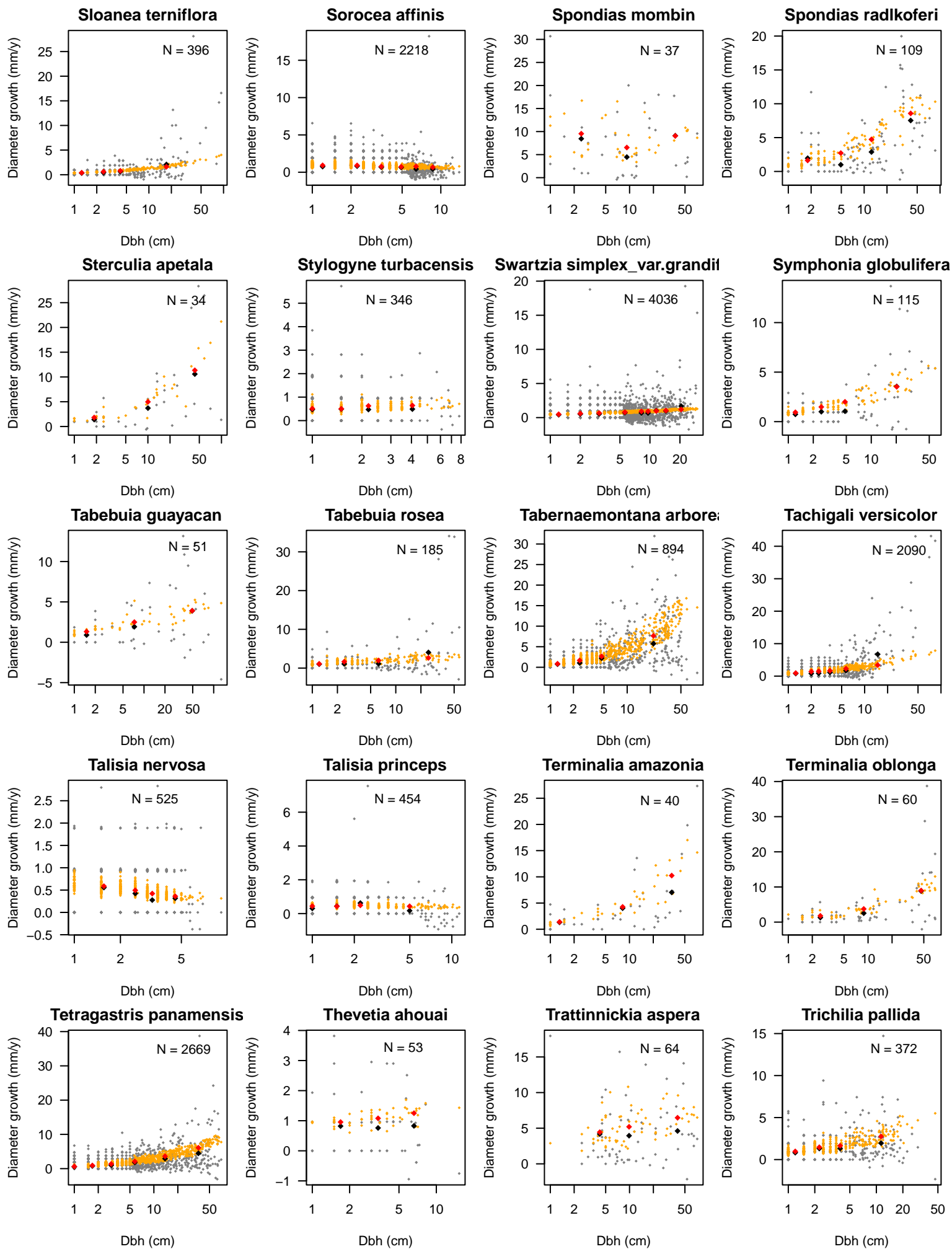

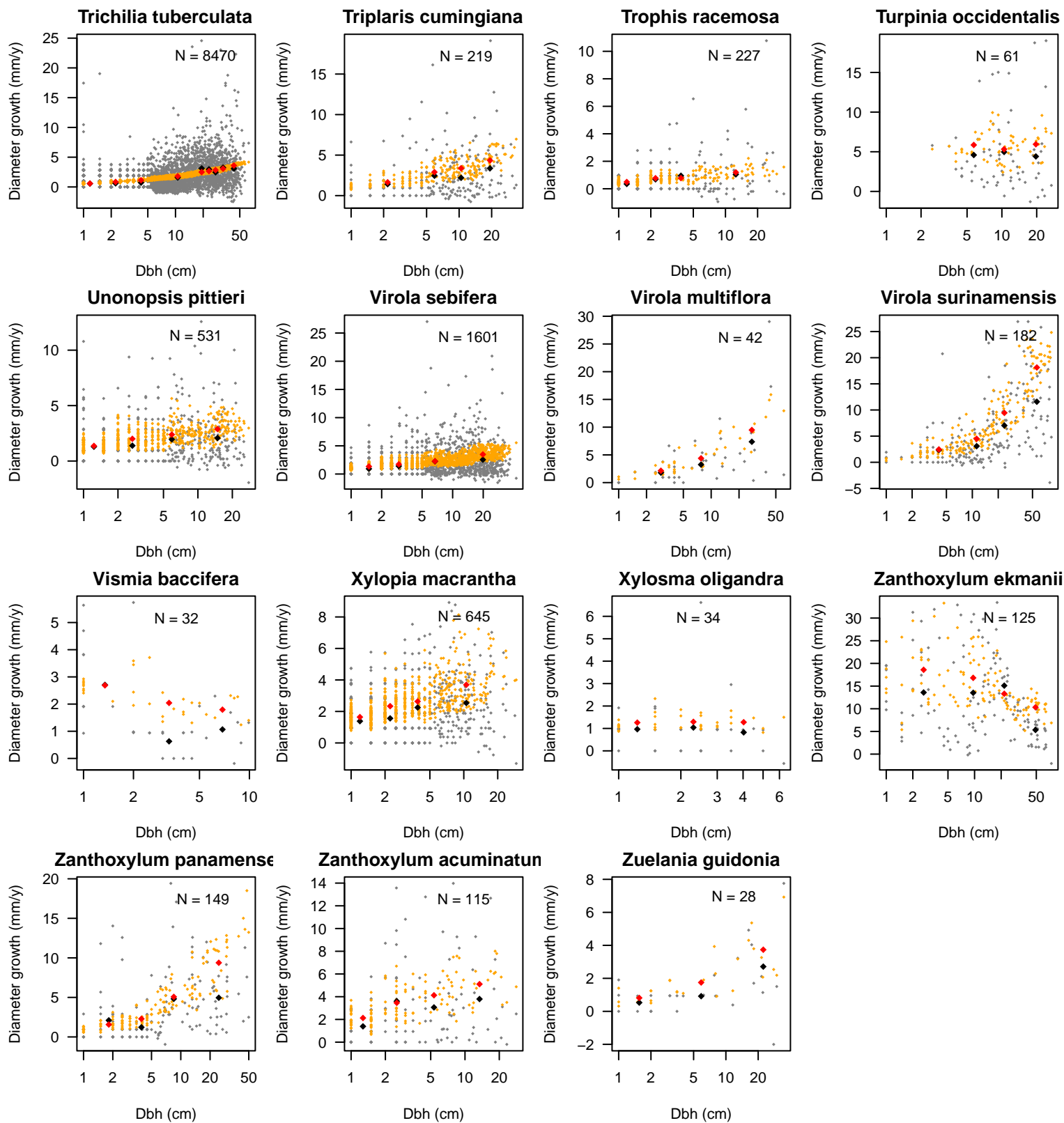

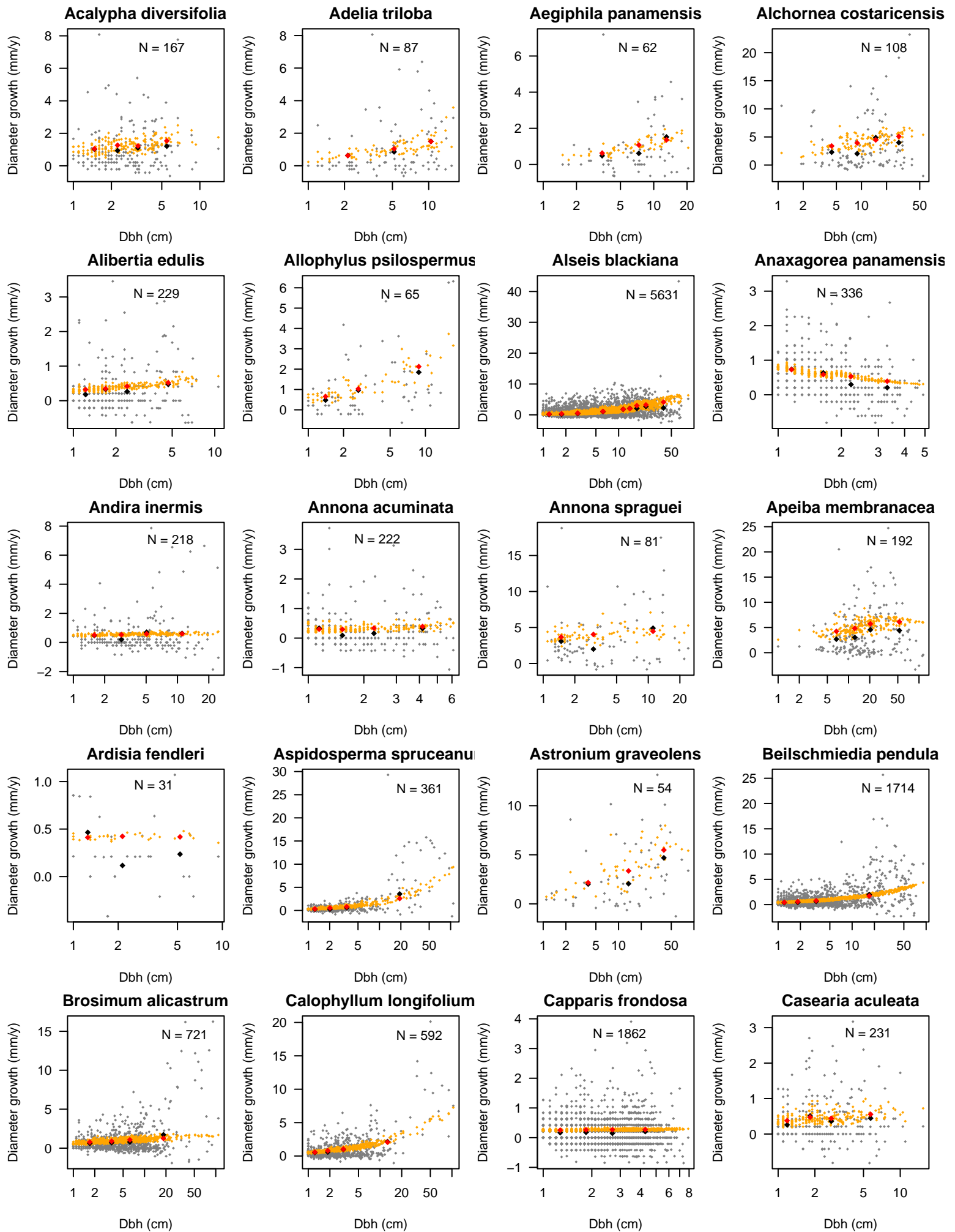

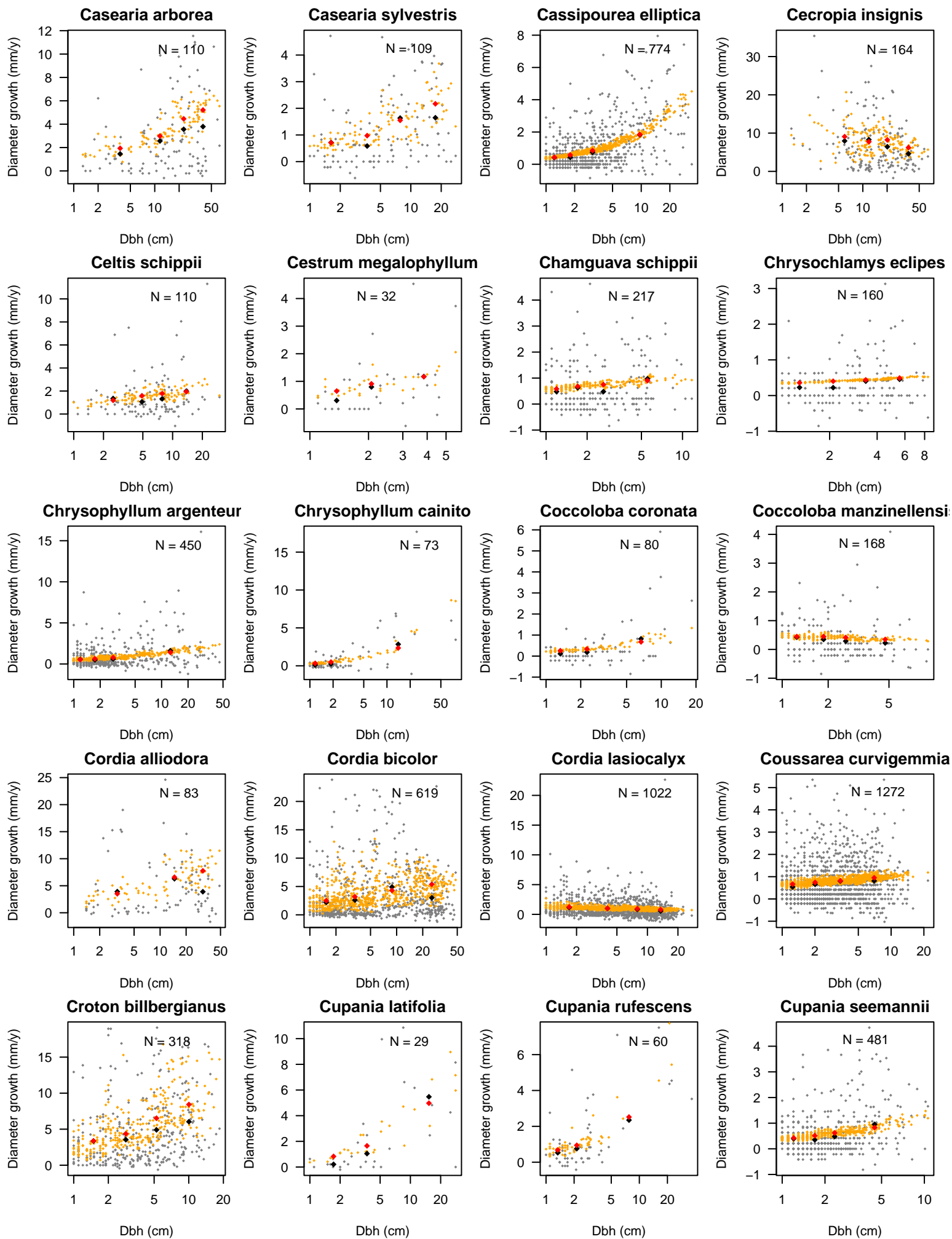

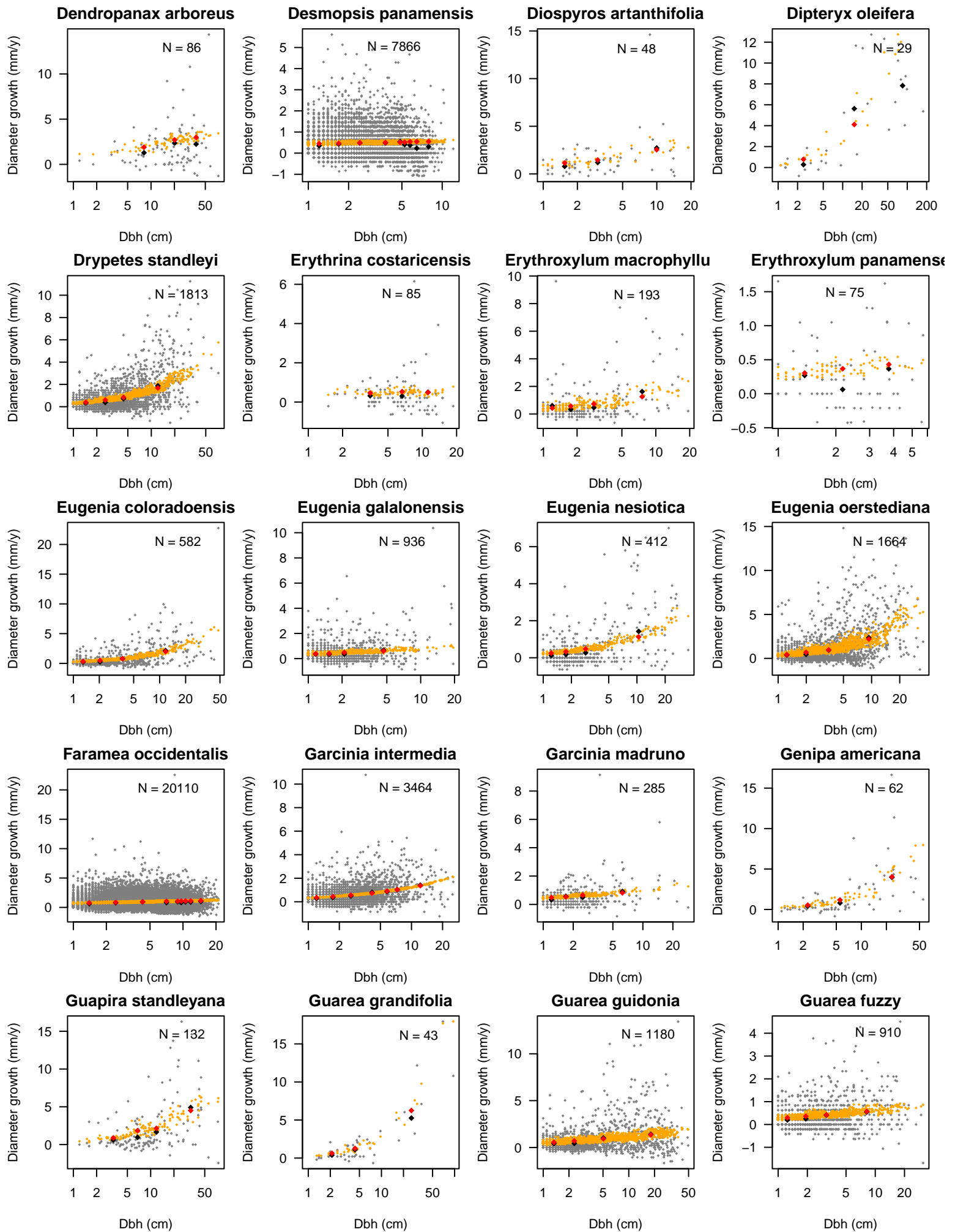

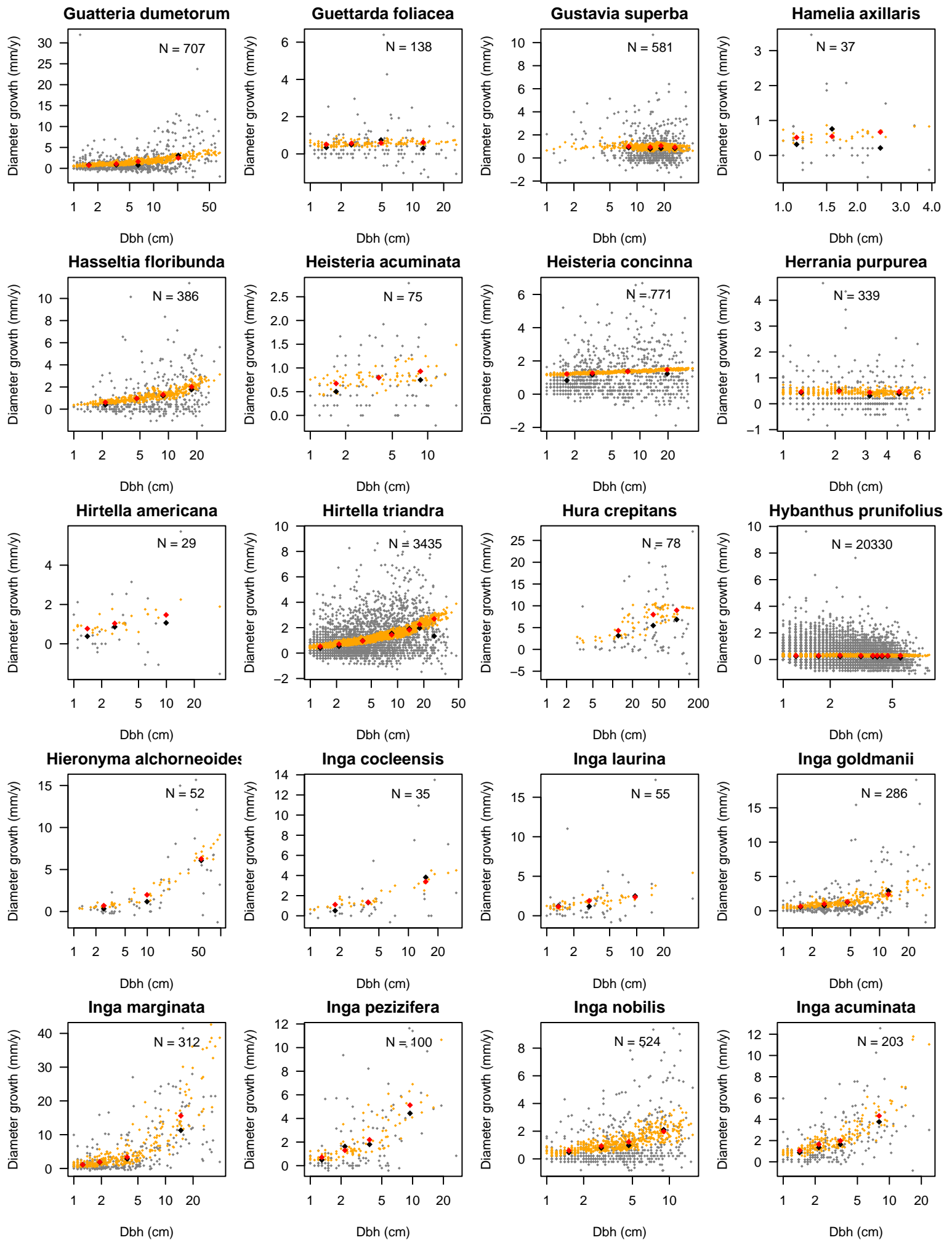

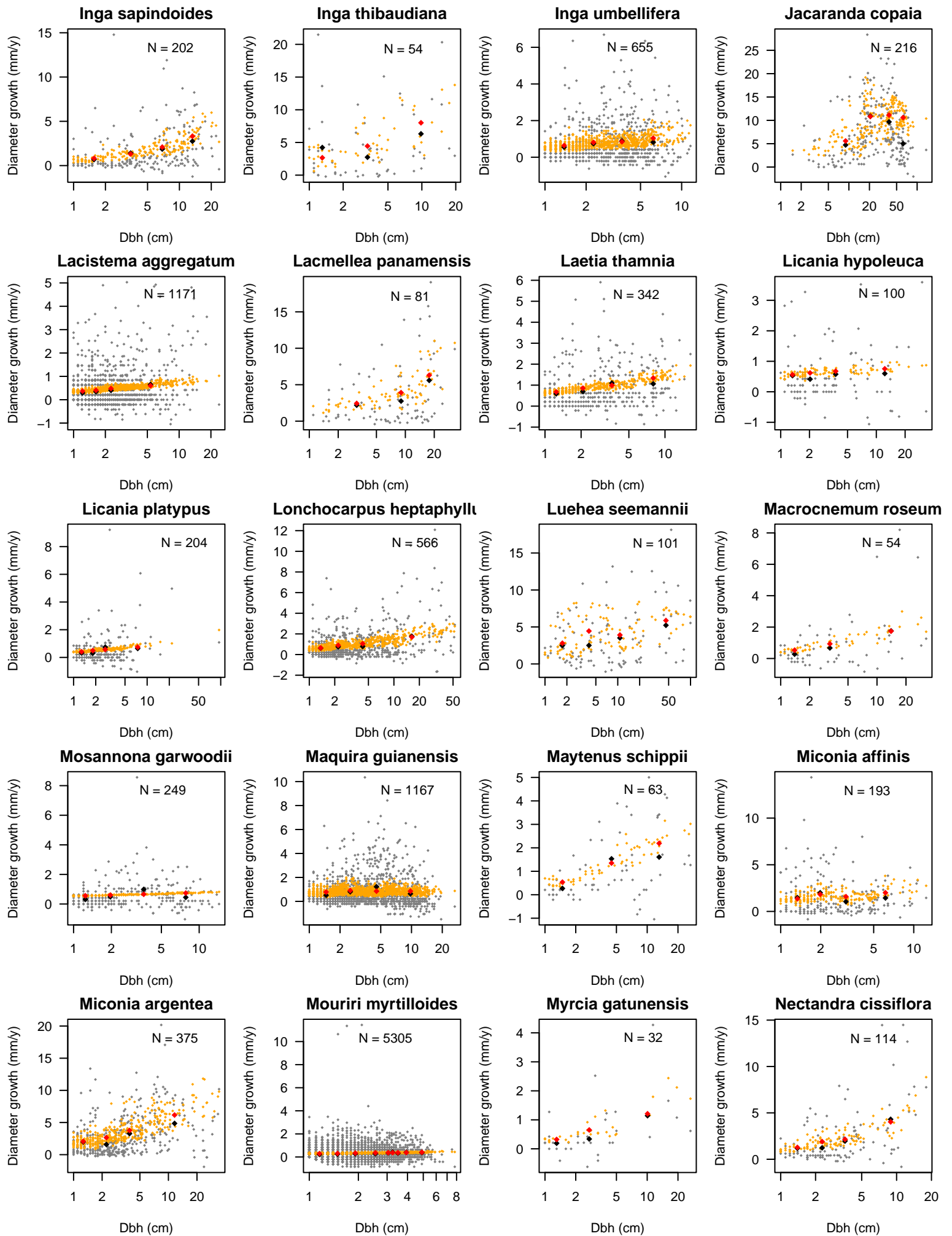

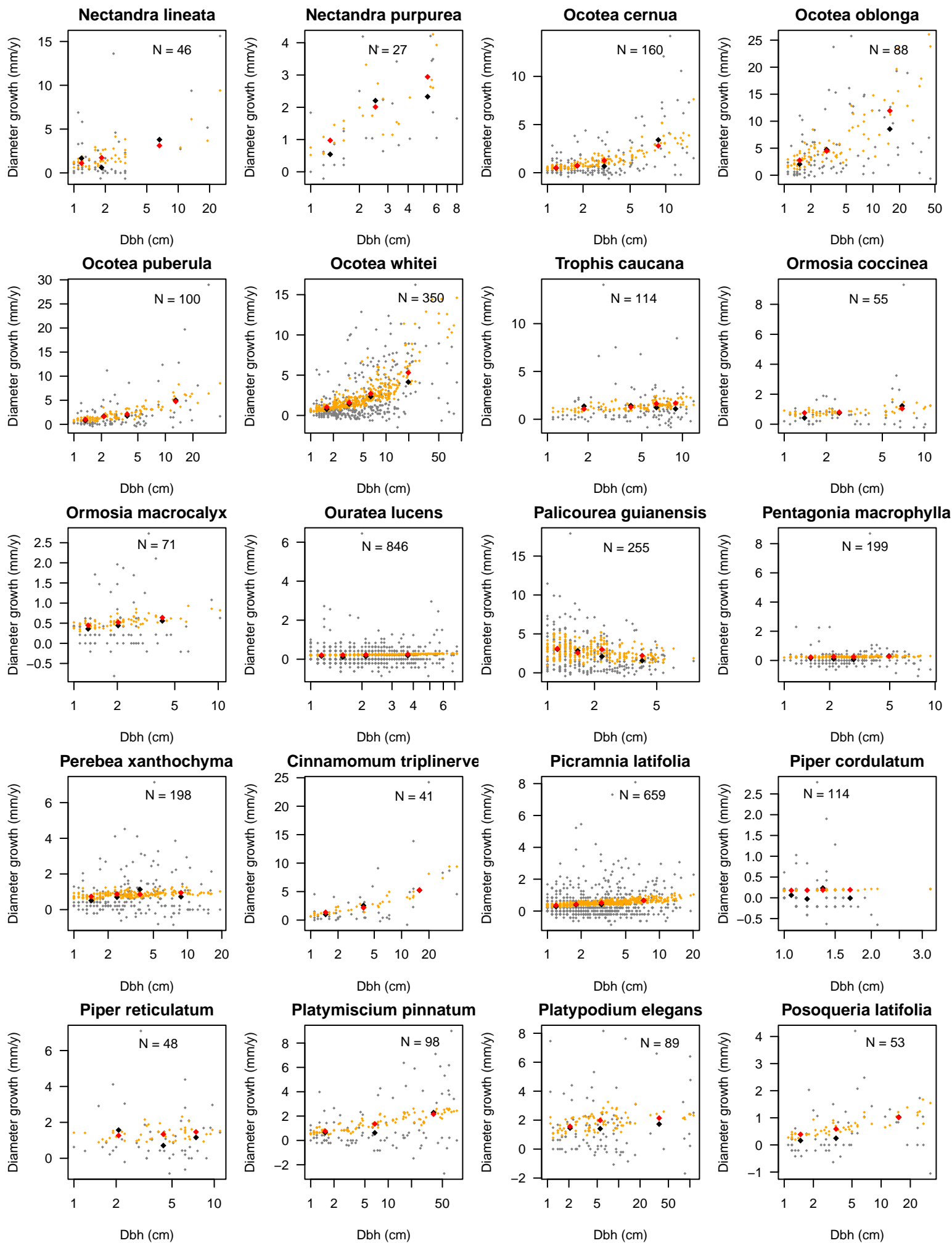

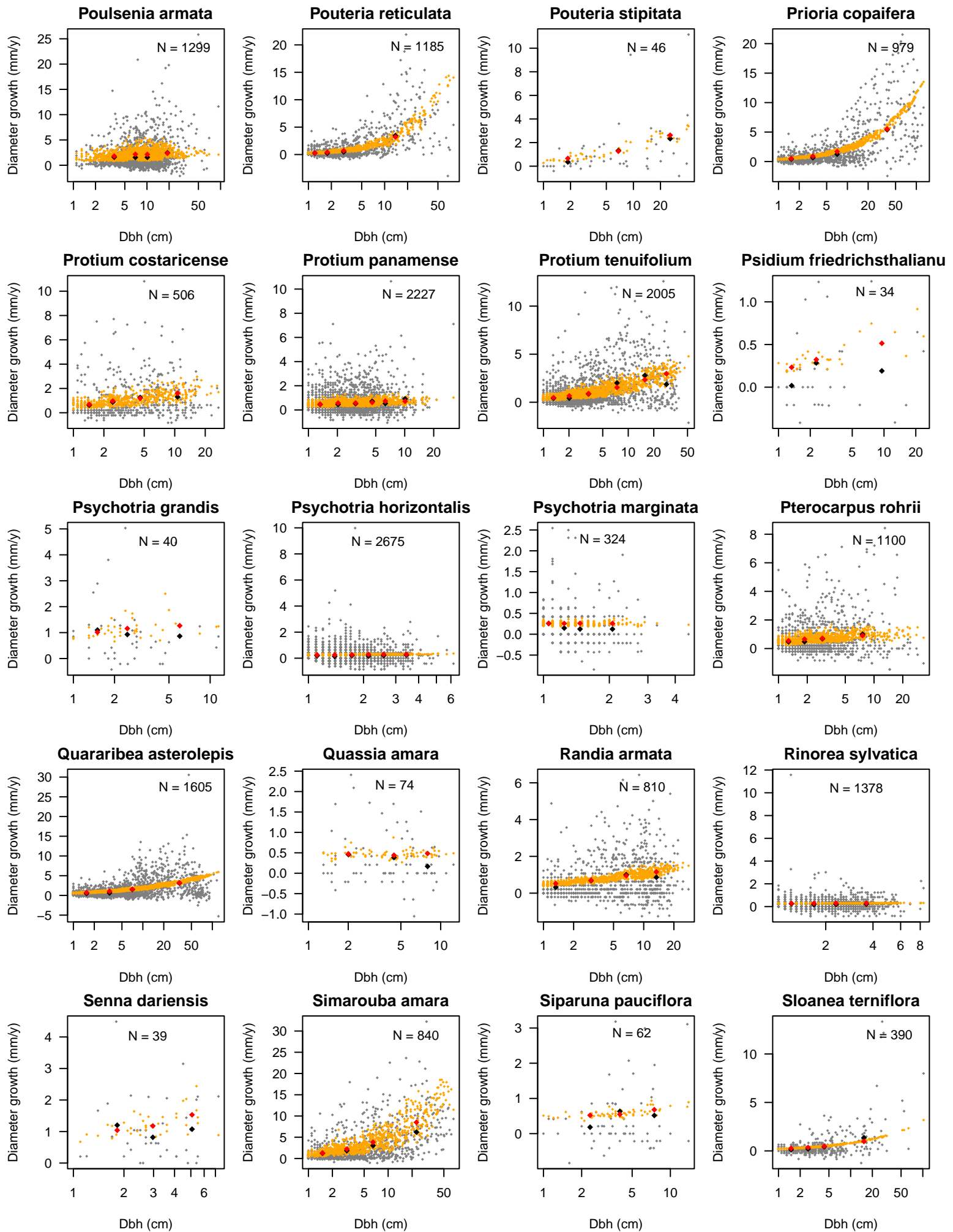

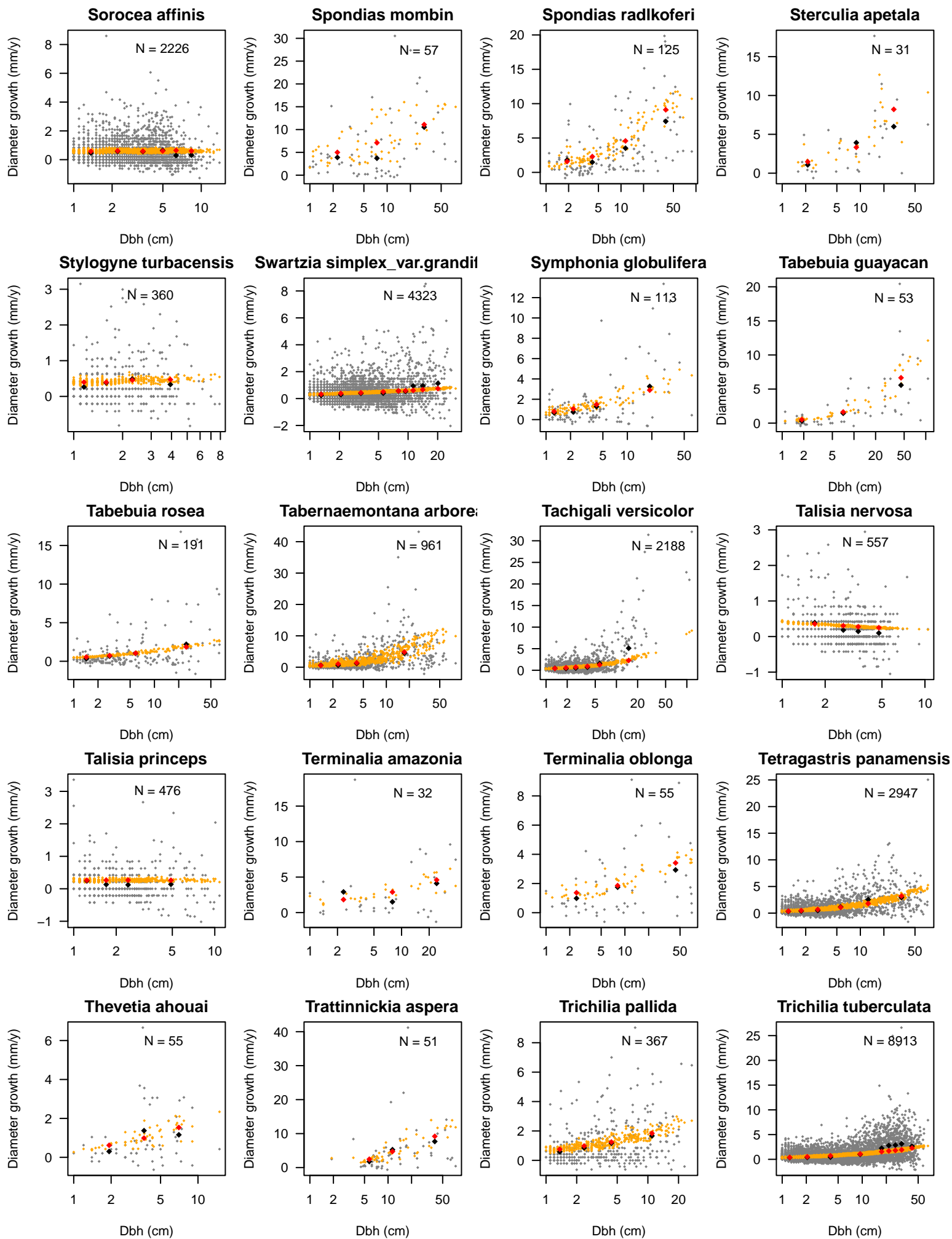

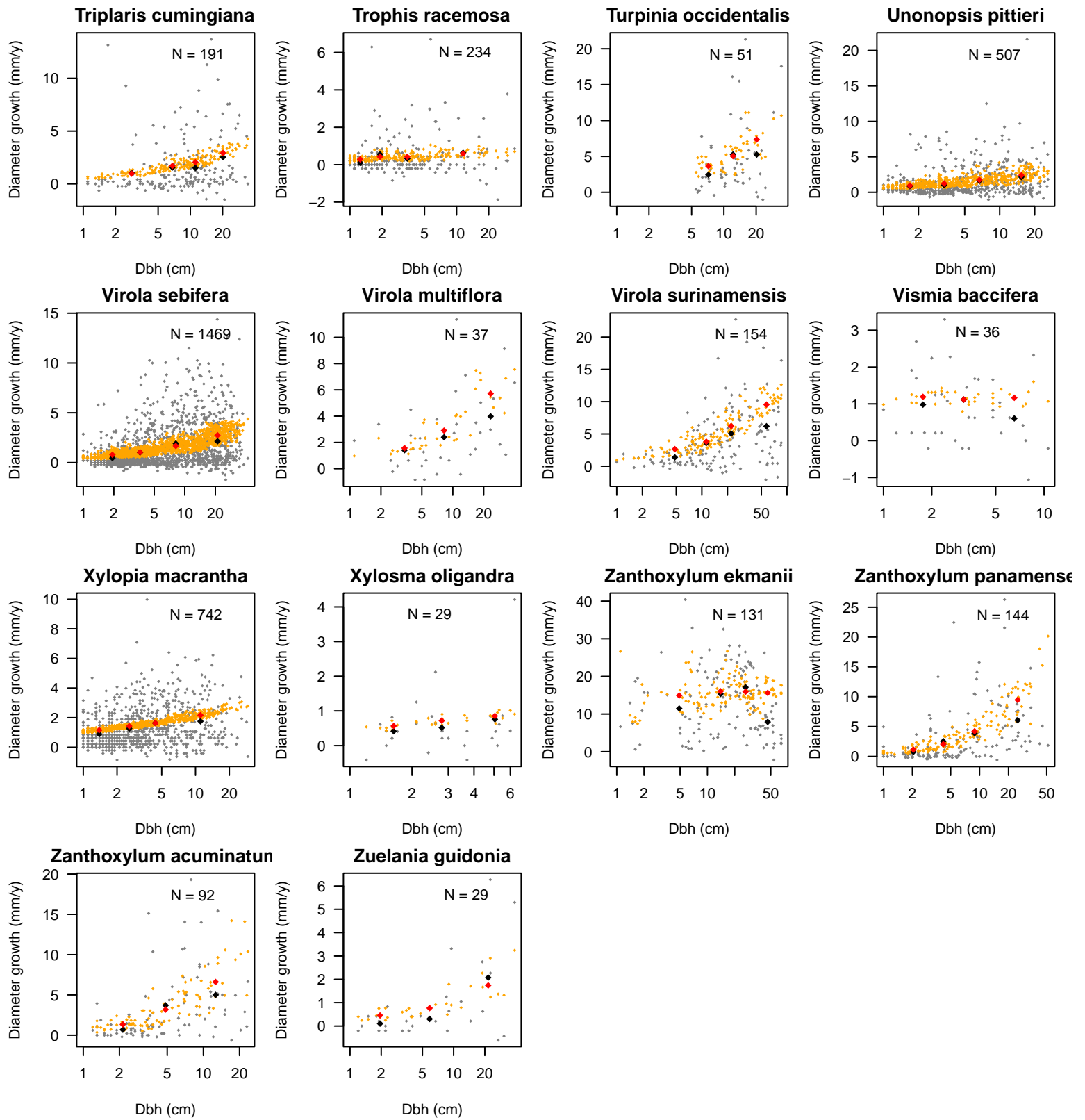

Supplement: Figure S3 — Dbh dependence of growth rates for species with ≥25 individuals in the two census intervals (1985−1990, 1990−1995). Observed and predicted growth rates of individual trees are displayed as grey and orange dots, respectively. Mean observed and predicted growth rates in different size classes are displayed as black and red dot, respectively. For species with <100 individuals, the dbh range was split into three size classes each containing a third of the individuals. For species with <2000 individuals, four size classes contain 25% of the individuals each. For species with <3000 individuals, six size classes contain 25%, 25%, 25%, 15%, 5% and 5% of the individuals, respectively. For species with <4000 individuals, seven size classes contain 25%, 25%, 25%, 15%, 3.3%, 3.3% and 3.3% of the individuals. For species with ≥4000 individuals, nine size classes contain 25%, 25%, 25%, 15%, 2%, 2%, 2%, 2% and 2% of the individuals. We only plot the size dependence for species with a maximum dbh of >3 cm. Predicted mean growth rates were calculated at mean observed light level and mean dbh of the individuals in the respective size class. (PDF) [file pone.0025330.s003.pdf]
